# The Molecular Machinery of Synaptic Plasticity and Its Potential Role in the Aetiology of Schizophrenia

**DOI:** 10.1007/s12035-026-05954-1

**Published:** 2026-07-02

**Authors:** Brian J. Morris

**Affiliations:** School of Psychology and Neuroscience, College of Medical, Veterinary and Life Sciences, University of Glasgow, Glasgow, G12 8QQ UK

**Keywords:** Schizophrenia, Psychosis, Synapses, Genetic association, Parvalbumin, Pleiotrophin

## Abstract

Altered glutamatergic and dopaminergic transmission in regions including cortex and hippocampus is thought to contribute to schizophrenia symptoms. The prominent role of glutamate (particularly via NMDA receptors) and dopamine (particularly via D2 receptors) in synaptic plasticity, and the impairment of plasticity-associated cognitive function in the condition, has suggested that schizophrenia may be viewed as a disorder of synaptic plasticity. This is encouraging, as regards developing improved treatments, as plasticity by its nature is dynamic and malleable. However, there are many distinguishable forms of synaptic plasticity, and it is not immediately obvious whether all forms are affected, and throughout the brain, or whether specific forms of plasticity are compromised, and only in certain brain regions. Here, I describe the molecules mediating various forms of plasticity, and collate the electrophysiological, imaging, pathological, genetic and biochemical evidence to address their possible dysfunction in schizophrenia. The overall picture is consistent with suboptimal function of all forms of plasticity, in circuitry centred on prefrontal cortex and thalamus. Many of the neurobiological changes characteristic of schizophrenia (reduced metabolic activity, GABAergic interneuron gene expression and dendritic spine density, in circuitry centred on prefrontal cortex) can be viewed as consequences of compromised plasticity rather than fundamental aetiological factors. Of hundreds of genes potentially contributing to genetic risk, more than 60 are directly implicated in plasticity processes, comprising receptors, voltage-sensitive Ca^2+^ channels, scaffold proteins, GTPases and kinase cascades. The conclusion is that multitudinous mechanisms of plasticity are all likely to be implicated in schizophrenia aetiology, but only in discrete neural circuits.

## Introduction

Schizophrenia is an extremely debilitating disease with a high prevalence and a poor prognosis. Schizophrenia typically appears during late adolescence/early adulthood, and then follows a chronic course characterised by recurring positive symptoms (e.g. hallucinations and delusions) and continual negative symptoms (e.g. anhedonia, avolition, social withdrawal and self-neglect) and cognitive deficits (e.g. inability to sustain attention, loss of executive function). The ability to control positive symptoms in many patients (at least in the short term) using dopamine antagonist drugs has dramatically reduced the need for long-term institutionalisation. However, negative and cognitive symptoms remain resistant to existing drugs, and a generally insurmountable barrier to long-term recovery.

The last three decades have seen substantial advances in our understanding of the neuroanatomical and genetic basis of schizophrenia and related diseases [1–3]. It has become clear that there is a major genetic contribution to disease risk. Other major risk factors, reflecting environmental influences, include prenatal maternal (severe) stress or infection, perinatal trauma or hypoxia and heavy cannabis use in adolescence/young adulthood [2, 4]. While understanding of the root causes of the neural dysfunction in schizophrenia has grown, it has not been matched by understanding of exactly how the neural circuitry is compromised.

Synaptic plasticity is crucially important to optimum function of CNS synapses. The maintenance of synaptic efficiency at optimal levels, across a range of inputs with differing levels of activity, and across time, is a highly complex and sophisticated process. It requires a large number of molecular components to operate together in synchrony, like cogs in a precision timepiece, or like the various musical instruments in an orchestra. Substantial dysfunction of a single element, or subtle dysfunction of many elements, will inevitably result in loss of harmony and disintegration of performance. The delayed response of positive symptoms to dopamine antagonist drugs raised the possibility that the therapeutic actions might involve a slowly developing modulation of the operational effectiveness of neural circuitry, or functional plasticity. Dopamine pathways are well-known as modulators of synaptic plasticity [5, 6]. Considering the importance of signalling via the NMDA class of glutamate receptor (NMDA R) for many forms of synaptic plasticity [7, 8], the concept that schizophrenia might be usefully viewed as a disorder of synaptic plasticity was then strengthened by multiple lines of evidence implicating hypofunction of NMDA Rs in the neurobiology of schizophrenia (discussed below). This idea has gained traction in recent years, and yet a comprehensive and critical examination of the underlying evidence has been lacking. The following sections address the multidisciplinary evidence that synaptic plasticity might be compromised in schizophrenia, and then focus on the extent to which any impairments might be viewed as causal for the condition, or consequential from other core neurobiology.

### Neuroanatomical Basis of Schizophrenia

Converging neurochemical, in vivo imaging and psychological data have provided substantial support for the view that the primary neural circuitry affected in schizophrenia centres on prefrontal cortex (PFC), thalamus, hippocampal formation (HF) and regions to which they are directly connected [9, 10].

- Prefrontal Cortex and Hippocampus

The most robust imaging phenotype detected in people with schizophrenia is reduced metabolic activity in PFC (hypofrontality) [11–22]. The nature of this hypofrontality is discussed in more depth in the section below on “Regional metabolic activity”.

The magnitude appears to correlate with the extent of negative and cognitive symptomology [11, 13, 14, 18, 22–28]. Indeed, the characteristic “cognitive” symptoms of the disease are probably closely related to the negative symptoms [29–31]. Of the negative symptoms, such as avolition, anhedonia and low mood [32], motivation and pleasure sensation are usually linked to ventral tegmental area (VTA) dopamine pathways to ventral striatum/nucleus accumbens (nAcc) and PFC [31].

With regard to the neural circuitry involved in delusional thought processes, PFC and orbitofrontal cortex regions are believed to be prominently involved [33–35]. Hence PFC dysfunction is implicated in positive, negative and cognitive symptoms [9].

Equally, the most robust neurochemical finding in schizophrenia is reduced expression of GABAergic interneuron markers—parvalbumin (*Pvalb*), somatostatin (*Sst*), calbindin (*Calb1*) and GABA synthetic enzymes (e.g. GAD67)—in PFC and HF [36–46]. This anatomical locus is entirely consistent with the cognitive impairment characteristic of schizophrenia, which is most readily observed in domains that involve HF/PFC circuitry, such as working memory, executive and attentional function [17, 47, 48].

b. Thalamus

**Fig. 1:**
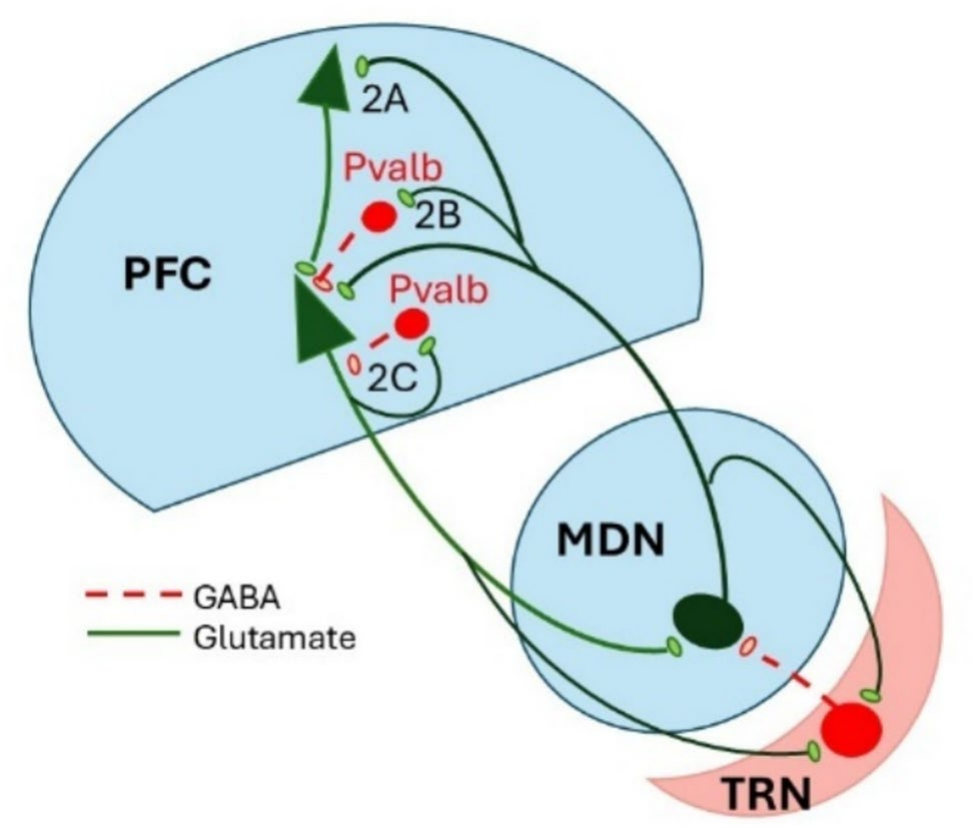
Schematic diagram of relationship in PFC between glutamatergic pyramidal cells in cortex, afferent input from thalamus (2A), and Pvalb + basket cells (2B) or axo-axonic cells (2C). Synapses 2 A and 2B are equivalent to those shown in more detail in Fig. 2

Evidence supports the intuitive expectation that the PFC and HF are not affected in isolation, but rather as part of dysfunction in limbic thalamocortical (TC) circuits. Along with reduced PFC metabolic activity, imaging studies also report reduced activity in schizophrenia of the mediodorsal nucleus of the thalamus (MDN) [49, 50], which provides the major thalamic input to PFC. Neurochemically, cortical Pvalb + interneurons are closely related to the neurones of the thalamic reticular nucleus (TRN), which are also GABAergic with high levels of Pvalb (and also voltage-dependent K ^+^ channel genes such as *Kcnc1*, which enable rapid spiking). The TRN—a thin sheet of cells curved around the lateral and ventral aspects of the thalamus—sends dense inhibitory projections to all the other thalamic nuclei [51, 52]. Glutamatergic corticothalamic (CT) and TC projections both innervate the TRN via collaterals (Fig.1). Thus the TRN is strategically placed to modulate information flow between cortex and thalamus [53]. Collaterals from PFC and MDN innervate the TRN more densely than fibres from other cortical/thalamic areas [54], suggesting a particularly potent influence of TRN on PFC/MDN circuitry (Fig.1).

Functional imaging of people with schizophrenia during auditory hallucinations reveals activation of inferior colliculi, medial geniculate body of thalamus (also innervated by TRN) and temporal (auditory) cortex [55–58].

The TRN is proposed to act as an attentional gate [59, 60], or “attentional searchlight” [61], facilitating salient sensory stimuli and suppressing irrelevant stimuli. The TRN is also responsible for generating certain rhythmic oscillations, including γ oscillations associated with cognition, and sleep spindles associated with NREM sleep [62–65]. Modulation of TRN activity in mice is sufficient to produce schizophrenia-like changes in γ oscillations and sleep spindles [66]. Apart from innervating and inhibiting the other thalamic nuclei, the GABAergic neurones of the TRN also inhibit each other, restraining the suppression of other thalamic nuclei [67]. Reduced levels of intra-TRN inhibition lead to hyper-synchrony in TC networks [68].

Emerging evidence implicates TRN dysfunction directly in core neurophysiological changes in schizophrenia [69]. Thus patients exhibit disturbed γ oscillations [70] and sleep spindles [62, 71, 72], which are driven by TRN, correlating with working memory and sleep deficits respectively. Other characteristic cognitive deficits—such as impaired attention—are also predicted from TRN dysfunction early in the course of the disease. The positive symptoms of schizophrenia can also be readily equated with deficient thalamic filtering of sensory information, and thus with impaired modulation of TC feedback by the TRN [53]. Altered TRN expression of α7 nicotinic receptors and glutamate transporters (EAAT1-3) is reported in post-mortem patient tissue [73, 74]. Recent evidence also shows reduced *PVALB *expression in patients in TRN, just as in PFC [75], and reduced terminal GAD expression in MDN [37]. Indeed, cortical *PVALB *expression is regulated by TRN lesions [76], and reduced TRN function during development in rats profoundly affects PFC morphology [77]. Equally, transiently compromised activity in the MDN-PFC pathway during adolescence in mice has a long-lasting suppressive effect on PFC [78]. In fact, changes in MDN activity cause downstream alterations in PFC dopamine turnover [79], as do changes in TRN activity [80]. Hence the hypofrontality and PFC GABAergic deficits highly characteristic of schizophrenia [14, 81, 82] may be a consequence of TRN dysfunction. Note that these affected regions are all modulated by dopaminergic afferents from the ventral midbrain.

c. Accumbens/Ventral Striatum

While a considerable research effort has been directed at identifying functional dopamine alterations in dorsal striatum, due to the efficacy of D2 dopamine receptor antagonists in treating positive symptoms in many patients, the robust evidence for abnormalities is largely restricted to measures of dopamine activity in dorsal striatum, which are generally reported as elevated in people with schizophrenia [83–87]. More consistent with the profound changes in MDN-PFC connections is dysfunction of nACC. From structural imaging studies, volume of nACC is reportedly decreased in schizophrenia, whereas caudate (dorsal striatum) volume is not changed [88–90]. However, studies available to date do not support dopaminergic hyperactivity in ventral striatum [91], although interestingly there is now evidence suggesting DAergic *hypo*function in PFC [92–94].

There has been discussion over the role of aberrant plasticity in the cascade of neurobiological events leading to schizophrenia symptoms [95–99]. The core circuitry showing altered function in the disease is fairly clear, as described above, but how strong is the evidence that plasticity is altered in this circuitry? The next sections consider the extent to which the biochemical and functional abnormalities detected in patients are consistent with aberrant synaptic plasticity.

#### Electrophysiological and Metabolic Evidence that Dysfunctional Cortical/Thalamic Synaptic Plasticity Exists in Schizophrenia

- Gamma Oscillations

Gamma rhythms are related to co-ordinated firing of populations of neurones, and are generated both internally within cortical and hippocampal areas, and are also driven to an extent by thalamic afferents governed by TRN firing patterns [63, 100]. Network γ oscillations are associated with plasticity processes, and directly facilitate plasticity, in particular, LTP [101–103]. One of the more robust observations in patients with schizophrenia is altered amplitude and power (a related measure) of γ oscillations [104–110]. The evidence is quite complex to interpret, due to the use of a variety of different stimulus conditions, recording methods and recording sites. However, there is a consensus that the amplitude and power of γ oscillations, when evoked by an auditory stimulus, are reduced in frontal and temporal cortex in people with schizophrenia [109, 111–115]. γ oscillations evoked by a cognitive task in frontal and temporal cortex are also reduced, although very high frequency γ oscillation amplitude may be increased in patients [109, 110, 116–118]. For resting state γ power, without the triggering auditory or cognitive stimulus, the evidence is mixed, with studies reporting that people with schizophrenia show reductions [119, 120], or no differences, or even increases [113, 121] in γ band power/amplitude. There is no obvious relationship between the nature/direction of the change observed and duration of disease, medication status, recording site or statistical power of the study, so the lack of a clear disease phenotype for resting γ oscillations makes its relevance to any impairment in synaptic plasticity more obscure. Importantly, though, for evoked oscillations, where a loss of power is robustly observed [109, 111–115], the abnormalities are observed in PFC but not in motor cortex [122], supporting a link to the core neurobiology of the disease. Furthermore, individuals carrying the 22q11.2 chromosomal microdeletion that greatly increases risk of schizophrenia also show decreased auditory-evoked γ oscillatory power [123], a phenomenon that shows a further suppression in those individuals with the microdeletion suffering with schizophrenia [124], and is also observed in a mouse strain that partly reproduces the genetic lesion [125].

Accepting the relationship between evoked γ oscillations and synaptic plasticity, the reduced γ oscillations in schizophrenia could be a primary neurobiological impairment leading in turn to a secondary deficit in plasticity processes. However, as will be discussed below, there is a great deal of evidence suggesting that impaired plasticity is a primary neurobiological impairment in the disease. In this case, can the reduced evoked γ oscillatory activity observed in schizophrenia potentially be explained as a secondary consequence/result of the primary impairment in plasticity processes?

Most forms of synaptic plasticity are initiated/triggered by activation of the NMDA Rs [7, 8], and so NMDA R antagonists reduce/prevent the induction of most forms of plasticity. However, acute blockade of NMDA Rs in humans leads to increased power and amplitude of evoked cortical γ oscillations [126, 127]. This would appear to be opposite to the robustly decreased evoked γ oscillations found in schizophrenia, even when measured under identical conditions [126]. For resting γ oscillations, where the nature of the change detected in patients is less clear, acute NMDA R antagonist administration also increases cortical γ band power/amplitude in humans [128–131].

Animal models could be very informative here. Indeed, NMDA R antagonists such as ketamine and PCP can disrupt γ rhythms in prefrontal cortex or hippocampal slices, although, in vivo, a main site of action outwith the cortex/HF, in TRN, is also plausible [53, 69, 72, 132]. This is likely to involve GluN2D subunit-containing NMDA Rs [133, 134], as the presence of this subunit decreases the voltage-dependent Mg^2+^block of NMDA R responses, allowing them to contribute to basal synaptic transmission [133]. In vivo in rodents*, *as in humans, acute NMDA R antagonist effects are clearly to enhance rather than suppress γ oscillations [135–140]. This appears to be true for both resting and evoked γ oscillations [139–142], and to be mediated via GluN2D subunit-containing NMDA Rs [143], although there are also a few reports of acute NMDA R antagonist suppression of evoked γ oscillations [144, 145].

However, chronic NMDA R antagonist administration in rodents causes a suppression of evoked (but possibly not resting) γ oscillations [125, 138]. This is interesting, as it implies that chronic rather than acute hypofunction at NMDA Rs may model the neurobiological dysfunction of schizophrenia, a theme that will recur in subsequent sections. This idea is supported by evidence that permanent deletion of NMDA R function (in Pvalb cells, including TRN) also decreases auditory-evoked γ oscillation amplitude [146], as in schizophrenia, although the observation that resting γ oscillation power was elevated is also interesting. Additional evidence that a primary deficit in plasticity mechanisms can be responsible for reductions in auditory-evoked cortical γ oscillation amplitude/power comes from a mouse model of the 15q13 deletion—a CNV which dramatically increases risk for a number of neurodevelopmental disorders including schizophrenia [147], and contains (among ~ 7 others) the *ARHGAP11B* gene that is involved in plasticity (see below). The mouse strain reproducing the microdeletion shows reduced auditory-evoked γ oscillation power [148]. Reduced γ oscillatory power is also reported in *Arc *knockout mice, which model the cumulative burden of numbers of common genetic risk variants, as well as addressing the function of this particular late-phase plasticity-mediator (see below) [149]. Hence reduced auditory-evoked cortical γ oscillation amplitude/power may be a consequence of compromised synaptic plasticity.

b. Cortical Long-Term Potentiation and Long-Term Depression

Based largely on studies in animal tissues, long-term potentiation (LTP) and long-term depression (LTD) have become well-characterised models of synaptic plasticity, where increased activity levels in afferent innervation induces long-lasting enhancement or suppression, respectively, of synaptic efficiency. The glutamatergic pyramidal neurones of the PFC receive a very strong innervation from the MDN, and from the HF, and both these pathways show LTP and LTD [150, 151]. Dopaminergic activity in VTA projections to PFC and HF also modulate plasticity, most prominently via D1-like receptors [5, 152, 153].

Several studies have addressed the possibility that cortical synaptic plasticity might be impaired in people with schizophrenia. Using transcranial magnetic stimulation (TMS) or transcranial direct current stimulation (tDCS), and measuring either EEG responses in motor cortex or somatic muscle responses, reduced plasticity (LTP) has been detected in people with schizophrenia [154–158]. However, there are some factors that should be considered. Firstly, all studies were focussed on motor rather than limbic circuitry, as equivalent studies on, say, prefrontal cortex, are extremely challenging technically. Yet there is little evidence from other approaches of substantial motor dysfunction in schizophrenia, and it was noted in the preceding section that deficits in γ oscillations in schizophrenia appear specifically not to involve the motor cortex. A recent study does however suggest impaired plasticity processes in schizophrenia in visual cortex [159]. Secondly, as pointed out in a meta-analysis which confirmed the overall impairment in plasticity in schizophrenia, the majority of schizophrenia subjects were taking anti-schizophrenia drugs at the time of the studies [160]. While there was no correlation between the degree of deficit and the levels of drug, and no detectable difference between medicated and unmedicated people where this was part of the study design, those studies were very likely underpowered statistically to detect such a difference. In fact, it is well-known that D2 antagonists suppress cortical and hippocampal plasticity in rodents [6, 161–163] and humans [164]. This potential confound has yet to be refuted.

It has been proposed that deficits in LTD can be detected in motor cortex in patients with schizophrenia [158]. The same reservations apply as with the assessment of LTP; however, this may be a hint that it is not just a single form of plasticity that is functioning sub-optimally.

c. Ventral Striatal (nACC) Plasticity

Synaptic plasticity in the striatum is best characterised in the dorsal part, as a component of basal ganglia circuitry. Here, D1 dopamine receptors play a role in the facilitation of LTP of glutamatergic synapses, whereas D2 receptors contribute to the induction of LTD [165]. The situation appears to be similar in the nACC, where NMDA Rs, mGlu5 Rs and D1 Rs are involved in LTP, although the contribution of D2 Rs to LTD may be less pronounced than in more dorsal striatum [166–168]. The possibility of altered synaptic plasticity in nACC in schizophrenia has not really been explored, although the levels of GAD appear to be reduced in post-mortem tissue from patients [169], and GAD expression is activity-dependent in nACC, as in other parts of the limbic system [170](discussed further below). In contrast, there may be an increase in the density of excitatory synapses onto spines in nACC, suggestive of enhanced afferent glutamatergic activity, although the diminished area of the post-synaptic density suggests the opposite [171]. LTD at PFC-nACC synapses will be facilitated by cannabis exposure [172], which is of note considering that high levels of cannabis intake during adolescence increase risk of schizophrenia [173]. It is very likely, considering the prominent role of dopamine receptors in this region, that nACC plasticity will be affected by long-term exposure to anti-schizophrenia drugs.

d. Regional Metabolic Activity

Reduced metabolic activity in PFC, as assessed by fMRI, or fluorodeoxyglucose PET imaging, is the most robust neurobiological observation in patients with schizophrenia. This metabolic “hypofrontality” is exacerbated by performance of a cognitive task requiring recruitment of PFC circuitry [11, 12, 14–17, 19–21]. There is evidence that the degree of hypofrontality correlates with the level of negative and cognitive symptoms [13, 18, 22, 26]. It is linked to reduced mediodorsal thalamic activity [49, 50], which also correlates with negative symptom severity [174]. There are also activity alterations in temporal lobe and connected thalamic regions. It seems that there may be decreases in resting activity, offset by elevated activity during hallucinations [13, 22, 82, 174–177].

It is worth mentioning here that there are some nuances to the observations of reduced prefrontal activity in schizophrenia. Increased activity relative to control subjects has been observed on a number of occasions, for example when the cognitive demands are very low, so that people with schizophrenia can complete the task adequately, albeit while inducing higher levels of prefrontal activation than control subjects. A component of this may be a reduced ability to de-activate the default mode network—a normal part of goal-directed cognitive engagement [178]. This is often described as an “inverted U” relationship between cognitive demand and prefrontal activation, with the curve shifted to the left in people with schizophrenia, such that there is a level of low cognitive load where they show higher activation than healthy controls, albeit showing the classic hypofrontality at higher levels of cognitive demand [179]. There are also the potentially confounding effects of anti-schizophrenia drug treatment to consider [180–184]. PFC hyperactivity has also been noted in first-episode people compared to chronic patients [185]. Nevertheless, the general picture is of reduced prefrontal activation in people with schizophrenia, compared to healthy controls, under conditions of significant but equal cognitive demand [12, 14, 21, 186].

Induction of LTP in HF induces changes in metabolic activity in PFC [187], so hypofrontality could be interpreted as evidence of reduced capacity for LTP in HF-PFC or thalamocortical afferents.

Clearly, suppression of network synaptic activity in neurons in vitro with tetrodotoxin (TTX) reduces metabolic activity [188]. Acute NMDA R antagonist (PCP or ketamine) administration in rats causes a rapid increase in PFC metabolic activity [189–195], but also produces a delayed hypofunction in PFC and thalamus [196]. Chronic PCP administration leads to hypoactivity in PFC, akin to the situation in schizophrenia [197]. It may be important that, in contrast to ketamine, PCP has a long time-course of presence in the plasma and in the CNS (in humans and rodents), in which case this may be further evidence that prolonged NMDA R hypofunction, outlasting the initial acute effects, is key for reproducing aspects of schizophrenia neurobiology in rodents [198]. Similar hypoactivity is observed in mice with reduced levels of the obligatory GluN1 subunit, where there is continual NMDA R hypofunction [199]. Hence hypofrontality can be interpreted as a consequence of sustained NMDA R hypofunction and compromised plasticity.

### Pathological and Biochemical Evidence that Dysfunctional Cortical/Thalamic Synaptic Plasticity Exists in Schizophrenia

- Dendritic Spines

Dendritic spines are the sites of excitatory synaptic transmission in many cell types. Spines can be highly dynamic structures (there is thought to be a relatively stable population, and a population that are less-constrained in terms of size and shape) [200]. Their length and number are maintained by AMPA R-mediated glutamatergic activity [201]. New spines grow in response to LTP/learning [202–207], and the size of existing spines tends to increase [203, 206, 208–212]. LTP induction also promotes stabilisation of dynamic spines [213]. Conversely, LTD is associated with spine shrinkage and elimination [214–218]. These structural changes in synapses are thought to be the most long-lasting sequelae of synaptic plasticity processes, and as such could be the most reliable indicator of altered plasticity in schizophrenia to be detected in post-mortem tissue. Decreasing network synaptic activity in cultured neurons with TTX reduces dendritic spine density [219, 220].

There is general consensus that the density of dendritic spines in cortex is reduced in schizophrenia. Reduced dendritic spine density in prefrontal and other cortical areas (including visual and auditory cortex) in patients with schizophrenia has been consistently observed [221–228]. A similar reduction in spine density has been noted within HF [229]. Analysis of spine size/morphology is rare, presumably reflecting technical difficulties with post-mortem tissue. However, Sweet, Lewis and co-workers report that it is particularly the density of small spines that is reduced in schizophrenia [224, 226]. The evidence is certainly consistent with impaired glutamatergic synaptic plasticity. The prevailing interpretation, however, has focussed on possible effects early in development, due to poorly controlled synaptic pruning by microglial cells. However, since spine number in the mature brain is affected by the recent history of activation by the afferent innervation, and alters with synaptic plasticity, we can speculate that the reduced spine density is a lasting trace of reduced levels of LTP-like plasticity. However, it should be noted that the evidence is equally compatible with enhanced LTD, and further, that it is not completely clear that spine density and size is unaffected by chronic dopamine antagonist exposure, an ever-present difficulty with post-mortem tissue [230, 231].

The concept that spine density reductions may reflect plasticity-related phenomena rather that early developmental dysfunction is supported by preclinical evidence from the use of NMDA R antagonists to suppress plasticity. In adult rodents, acute NMDA R antagonist administration rapidly increases PFC spine density [232–236], likely due to a period of enhanced glutamate release. However, chronic antagonism decreases spine density below basal levels, while sustaining the suppression of LTP [237–239]. Similarly, mice with genetic deletion of NMDA R subunits show impaired LTP and LTD, and reduced dendritic spine density in cortex and HF [240–242]. This implies that, for reproducing schizophrenia-like neurobiological changes in rodent models, chronic NMDA R antagonism/loss-of-function is far more relevant than acute antagonism, a theme which has already been introduced, and will recur in subsequent sections.

The microtubule-associated protein MAP2 is a key contributor to microtubule dynamics. Activity-dependent induction of *Map2 *mRNA seems to be a crucial part of morphological changes in dendritic spines during late-stage plasticity [243]. Its expression in neurons is unusual, in that, as also with CamKIIα, the mRNA is present at high levels sub-synaptically in dendrites, as well as in the cell soma, presumably to allow more rapid activity-dependent modulation of protein synthesis. Indeed, *Map2 *mRNA expression is highly sensitive to the level of glutamatergic transmission, and is increased specifically in dendrites by induction of LTP [244-246]. MAP2 protein is selectively decreased in prefrontal and auditory cortex, but not occipital cortex, in patients with schizophrenia [224–226, 247, 248], although this is less clear in the hippocampal formation [249–251]. The effect in PFC is quite dramatic, with immunoreactive signal reduced by ~ 50% in some reports [247, 248], and may occur in parallel with a loss of complexity in dendritic arborisation [223, 252, 253]. There is no evidence for the *MAP2* gene contributing any genetic risk of schizophrenia, but the overt reduction in *MAP2* expression in schizophrenia is therefore suggestive of reduced levels of plasticity, and may be related to the reductions in dendritic spine density.

There is an interesting comparison to be made with CamKIIα expression, since dendritically localised *Camk2a *mRNA is also elevated after LTP induction [244, 246, 254]. Expression of mRNA encoding CamKIIα, however, appears not to be decreased in schizophrenia; it may even be slightly increased relative to controls [255]. Hence while the reduced dendritic *MAP2* expression is suggestive of impaired plasticity, this is not supported by the lack of reduction in *CAMK2A* mRNA expression. That said, there appears to be no information on the levels of CamKIIα protein expression in schizophrenia, and the observed reductions in Map2 expression were all detected at the protein level. Equally, during LTP, Map2 expression is more strongly regulated via cAMP as compared to Ca^2+^signalling, whereas the opposite is the case for CamKIIα expression [256]. The decreased expression of Map2, but possibly not CamKIIα, in schizophrenia could therefore be interpreted in terms of a particular impairment in cAMP-dependent pathways.

b. Glutamatergic Synapse Dysfunction

The reduced density of dendritic spines is strongly suggestive of impaired function of glutamatergic synapses. Other evidence is consistent with this. Glutamate levels, as assessed by magnetic resonance spectroscopy (MRS), are often found to be decreased in PFC and possibly increased in thalamus in people with schizophrenia [257–260]. While this is suggestive of reduced prefrontal glutamatergic activity, it should be remembered that MRS measurements are quite a crude index of neurotransmitter activity, as volume resolution is low, and total tissue content of metabolite is measured, meaning that transmitter glutamate is not discriminated from metabolic glutamate [261, 262].

A variety of signalling proteins present in biochemically isolated post-synaptic densities are present in lower levels in tissue from patients compared to controls [263]. Meta-analysis confirms a decreased number of post-synaptic elements, as well as dendritic spine density, in post-mortem PFC tissue from patients with schizophrenia [228].

*N*-acetylaspartate (NAA) is the precursor of *n*-acetylaspartylglutamate (NAAG), argued to be an agonist at the mGlu3R and able to suppress HF LTP [264]. NAA levels may provide some kind of index of the level of glutamatergic synaptic activity. People with schizophrenia exhibit robust reductions in PFC and thalamic NAA, but not striatal NAA [258, 265–270]. Also, chronic ketamine users develop reduced NAA levels in the thalamus [271], as do people with anti-NMDA R encephalitis [85], alongside the development of schizophrenia-like symptoms. Equivalent decreases are seen in rat temporal cortex after chronic PCP [272], so NAA levels may be a useful translational marker of NMDA R hypofunction, and the reduced PFC/thalamic NAA in schizophrenia may be indicative of suppressed activity in corticothalamic loops.

c. Molecular Markers of Plasticity

Arguably the most robust molecular marker for synaptic plasticity is the immediate-early gene *Egr1 *(a.k.a. zif268, Ngf-Ia or Krox24) [254, 273–277]. Transcription of *Egr1 *in the post-synaptic neurons is elevated following LTP induction in HF synapses [254, 273, 276] and thalamocortical synapses [278, 279]. Indeed, constitutive cortical *Egr1 *levels appear to be a reliable proxy marker indicative of recent glutamatergic activity [275, 277]. Suppressing network activity in cultured neurons with TTX reduces *Egr1 *expression [280]. As with PFC metabolic activity [196], acute NMDA R antagonist (PCP) administration in rats causes a rapid increase in *Egr1* expression, followed by a remarkably prolonged (> 48 h) suppression below basal levels, in PFC and HF [281, 282]. This most likely reflects the suppression of afferent glutamatergic activity. Consistent with compromised plasticity in schizophrenia, *EGR1 *expression is indeed robustly decreased in PFC from patients [283–286].

**Fig. 2:**
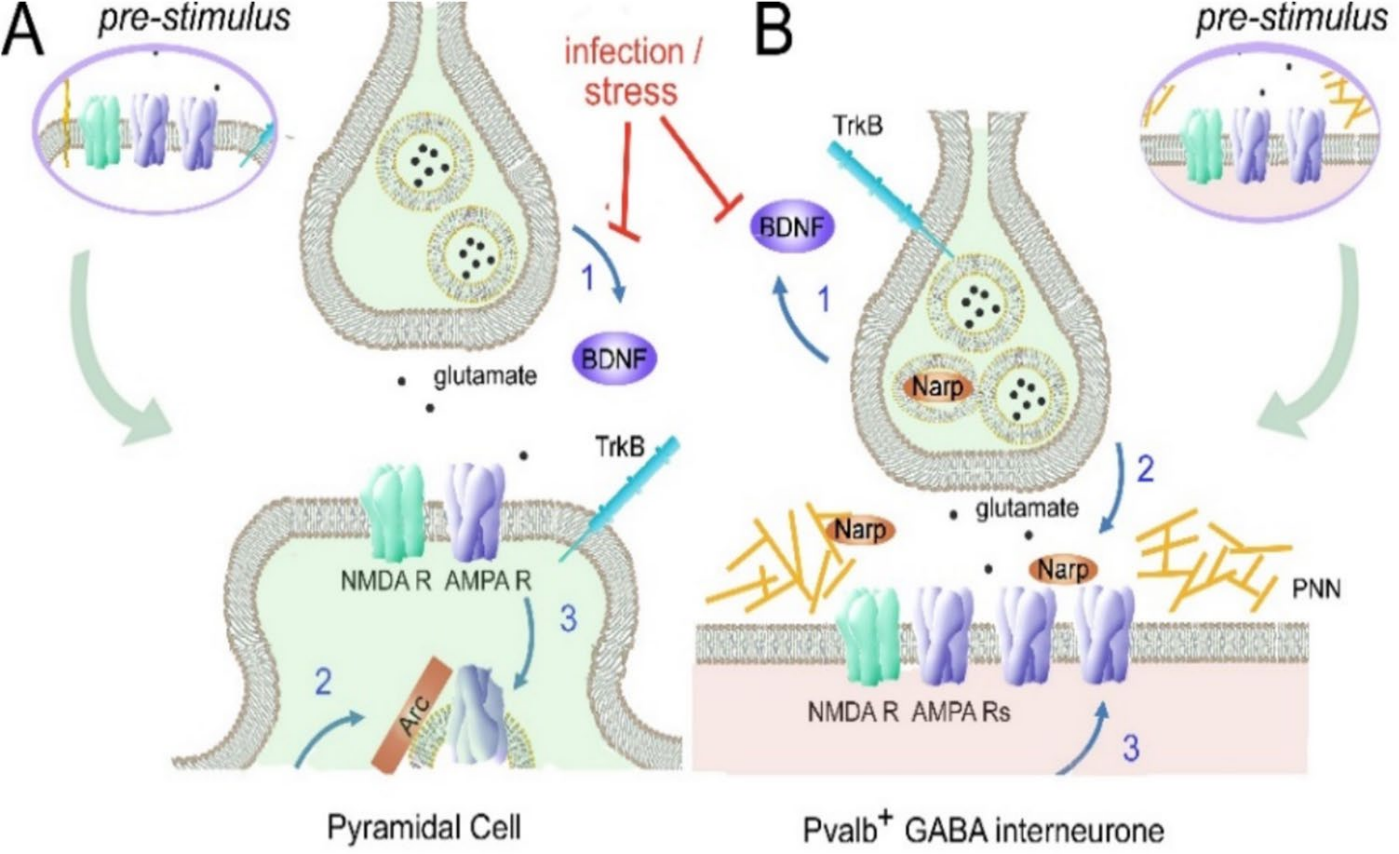
Down-scaling HSPlas mechanisms at excitatory synapses onto a glutamatergic cell (**A**) and Pvalb+ cell (**B**). Following increased levels of synaptic activity, at these or nearby synapses, BDNF is released (1), and stimulates rapid synthesis of post-synaptic Arc in the glutamatergic cells, and presynaptic Narp in the glutamatergic synapse onto the Pvalb+ cell (2). Arc then facilitates AMPA R endocytosis, while Narp is released and facilitates clustering and local stabilisation of GluA4 subunit-containing AMPA Rs (3). The overall result is reduced synaptic AMPA R number at the synapse onto the excitatory neuron and increased synaptic AMPA R number at the synapse onto the inhibitory neuron. PNN = perineuronal net

There are two additional robust markers of recent activity at glutamatergic synapses [287–294]: activity-related cytoskeletal protein/activity-related gene 3.1 (Arc/Arg3.1), where mRNA is elevated post-synaptically in glutamatergic dendrites (e.g. A in Fig.2) after synaptic potentiation, and neuronal activity-related protein/neuronal pentraxin 2 (Narp/Nptx2)—synthesised pre-synaptically at glutamatergic synapses onto Pvalb^+^ cell dendrites (e.g. at B in Fig. 2) after synaptic potentiation. Arc and Narp are both expressed primarily in glutamatergic principle cells (Fig. 2) [292].

Arc suppresses the excitability of the glut-glutamatergic cell synapses, most notably by facilitating endocytosis of synaptic AMPA Rs [295, 296] via a protein complex [297, 297–299]. Conversely, Narp increases excitability of Pvalb^+ ^GABAergic cells, by enhancing synaptic clustering of AMPA Rs containing the GluA4 subunit [300], which are highly expressed in Pvalb^+^ cells (Fig. 2). This induction of Arc/Narp, and the corresponding suppression of the efficiency of excitatory synapses onto glutamatergic principle cells (Arc), and elevated activation of cells inhibiting glutamatergic principle cells (Narp), underlies a long-lasting, and not necessarily synapse-specific, form of plasticity, known as homeostatic plasticity (HSPlas). In this case, the increased expression of Arc/Narp during periods of heightened afferent activity serves to dampen the level of activation of the post-synaptic glutamatergic neurons (down-scaling) [301, 302]. A corresponding up-scaling form of HSPlas acts to facilitate glutamatergic projection neuron stimulation after periods of suppressed activity [303] (discussed further below).

*Arc *and Narp expression in the mature CNS is maintained by the level of synaptic activity [288, 291–293]. Enhancing network activity in neuronal cultures, via BDNF exposure, or via disinhibition due to blocking GABA_A_receptors (with subsequent release of BDNF) [287], increases *Arc* and *Narp *expression [287, 289, 290, 294] and expression of synaptic GluA4-containing AMPA Rs in Pvalb^+^cells [304]. Conversely, suppressing network activity in neuronal cultures with TTX reduces BDNF, *Arc* and *Narp *expression [287, 289], down-regulating GluA4-containing AMPA Rs in Pvalb^+^cells [304].

The role of Arc in Hebbian synaptic plasticity is difficult to separate from its role in HSPlas. While very clearly induced both post-transcriptionally and transcriptionally following LTP induction [305–308], it also plays a major role in post-activity down-scaling of synaptic function. Confusingly, expression of Arc is decreased in HF CA1 following induction of LTD [308], yet absence of Arc blocks LTD expression here [309].

It seems clear that expression of *NARP *[310–313], and possibly *ARC *[39, 311, 314], is decreased in PFC in schizophrenia; *NARP* by as much as 40%. Indeed, *NARP *expression has been proposed as a reliable schizophrenia biomarker [315].

If expression of Arc is suppressed or enhanced according to the recent history of local synapse-specific LTD or LTP respectively [308], it is interesting to consider that, in contrast to PFC, auditory cortex tissue from patients reportedly shows increased *ARC* mRNA levels (along with increased *EGR1 *mRNA levels) [316].

Decreased expression of *EGR1*, *ARC* and *NARP* in PFC in schizophrenia could be indicative of compromised (thalamocortical) synaptic plasticity, since expression of these genes is activity- (and plasticity-) dependent. We should remember, though, that these genes are all mediators of plasticity, and hence their decreased expression could equally well be the cause rather than the consequence of dysfunctional plasticity. In the case of *ARC*, as discussed below, the genetic evidence supporting a causal role for impaired ARC function in disease aetiology is strong, although this is not the case for NARP or EGR1.

d. GABAergic Interneuron Dysfunction

Thalamocortical synapse plasticity is particularly prominent early in development [317, 318]. The decline in the degree of plasticity as corticothalamic circuitry becomes fully developed may reflect the maturation of *Pvalb*+ ve GABAergic interneurons [319–322].

Reduced expression, at protein and mRNA level, of certain GABAergic interneuron markers (*Pvalb*, *Sst*, *Calb1*), in PFC and hippocampus, is the most robust neurochemical change reported in schizophrenia post-mortem tissue [36–46, 323]. Similarly, there are decreases in expression of GABA synthetic enzymes GAD67 (*GAD1*) (robustly observed) [38, 41, 283, 324–326] and GAD65 (*GAD2*) (less robustly observed) [324, 325, 327, 328]. The altered gene expression appears to affect both chandelier/axo-axonic cells and basket cells [36, 38, 39, 43, 46, 328–331]. Other classes of GABAergic interneuron in these regions appear to be little-affected.

*Pvalb *levels are known to be activity-dependent [180, 181, 332–335]. Like *Pvalb*, both GAD67 and GAD65 show activity-dependent levels of expression. Enhancing network activity in neuronal cultures, via BDNF exposure, or via disinhibition due to blocking GABA_A_ receptors (with subsequent release of BDNF) up-regulates expression of GAD67 (*GAD1*) and GAD65 (*GAD2*), while suppression of network activity (with TTX) leads to a compensatory reduction in expression [287, 290, 336–340]. Following acute NMDA R antagonist administration in rodents, there is a delayed (24 h) decrease in *Pvalb *expression [180, 181]. A similar suppression of *Pvalb *expression in PFC and TRN, but not in motor cortex, is observed with chronic NMDA R antagonist administration in rats [138, 197].

*SST *expression is probably also regulated by levels of afferent activity [243, 341, 342]. Hence rather than a generalised impairment of these interneurons, the reduction in expression of *Pvalb*/*Sst*/GAD67 (but not VIP or calretinin) [323, 343, 344] in PFC and HF from patients with schizophrenia probably reflects a selective loss of activity-dependent glutamatergic activity or plasticity.

While studies are sparse, it is interesting that reduced expression of GABAergic markers in SST + ve and Pvalb + ve interneurons is reported in visual cortex as well as PFC and HF [45, 345]. This is a similar picture to the loss of dendritic spines in schizophrenia, mentioned above, which is observed in occipital, visual and auditory cortex as well as in PFC and HF [221–229, 346].

As can be seen from the preceding discussion, many of the core neurobiological changes characteristic of schizophrenia, including metabolic hypofrontality, disrupted γ oscillations, reduced GABAergic gene expression and reduced dendritic spine density on cortical pyramidal neurons, can be viewed as consequences of compromised synaptic plasticity as easily as they can be viewed as fundamental changes resulting from adverse early developmental influences. The question then arises as to whether compromised synaptic plasticity can be a result of either genetic or environmental risk factors for the condition, and thus can be incorporated into theories of causation.

### Molecular Overview of Synaptic Plasticity Mediators

The processes of circuit formation during neurodevelopment, and of learning and memory formation or consolidation throughout life, rely on synapse-specific (Hebbian) plasticity. In many cases, this is mediated via the stimulation of NMDA class glutamate receptors (NMDA Rs). In contrast to AMPA class glutamate receptors (AMPA Rs), which mediate most basal fast excitatory transmission, NMDA Rs, in general, only become active with elevated pathway stimulation, leading to post-synaptic Ca^2+^influx, and long-term depression (LTD), or potentiation (LTP) [7, 347]. Note that stimulation of atypical Ca^2+^-permeable (GluA2 subunit-lacking) AMPA Rs is sufficient on its own to induce a form of LTD [8].

Kainate Rs also play a more subtle role in LTP and LTD, either as effectors of plasticity in the place of AMPA Rs, or as mediators of plasticity via AMPA R trafficking [348, 349]. In the latter case, sustained stimulation of kainate Rs containing the GluK2 subunit leads to downregulation of synaptic AMPA Rs (LTD), via suppression of protein kinase C (PKC) and cAMP-dependent protein kinase (PKA) [348, 349], while upregulation of synaptic AMPA Rs (LTP) can occur after transient kainate R stimulation [350].

Stimulation of metabotropic glutamate receptors can result in either LTP or LTD [351]. The Group 1 Rs (mGlu1 and mGlu5) seem to be required for the full expression of LTP and LTD induced by NMDA R stimulation or voltage-dependent Ca^2+^channel (VDCC) activation, and forms of both LTP and LTD can be induced by group 1 mGluR activation alone [8, 351, 352]. Of the Group 2 metabotropic glutamate receptors (mGlu2 and mGlu3), mGlu2 is mainly presynaptic, often suppressing glutamate release, and while mGlu3 is also often presynaptic, it can additionally be expressed by astrocytes and microglia [351]. Most evidence is consistent with the concept that mGlu2/3 activation is a component of LTD [351]. Group 3 receptors (mGlu4, 6, 7 and 8) are also frequently located pre-synaptically on glutamatergic terminals. Their effects on plasticity seem to be quite synapse-dependent, but generally suggest a role in LTD but not LTP [351]. Group 1 and Group3 Rs also contribute to depotentiation [351].

**Fig. 3:**
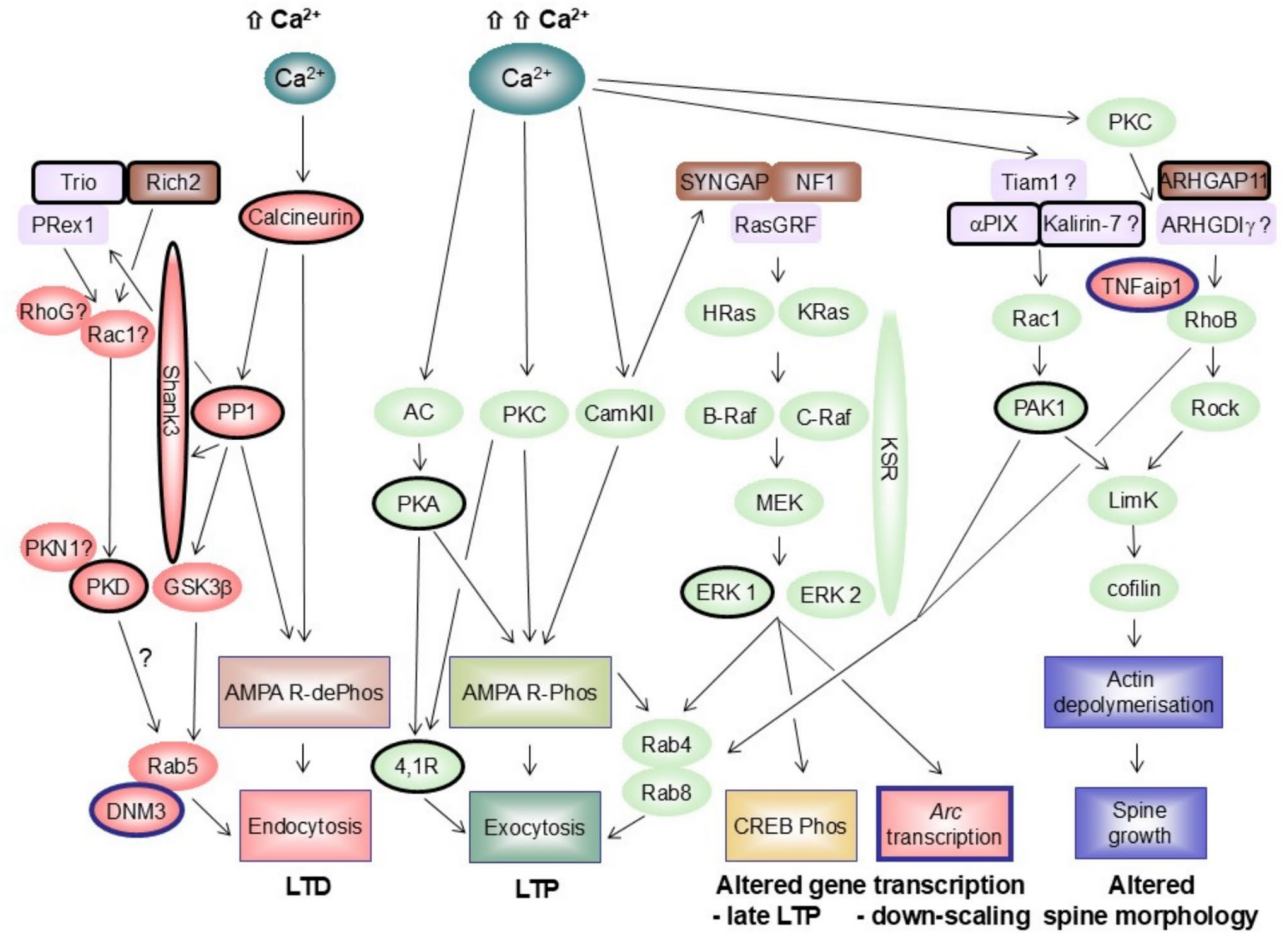
Homosynaptic plasticity: primary mediators. Bold outline for a symbol indicates that genetic evidence suggests a contribution to schizophrenia aetiology

The early phases of plasticity are sustained primarily by altered synaptic AMPA R levels—mainly containing GluA1 or GluA2 subunits, as regulated by phosphorylation or dephosphorylation of the subunit intracellular domains [353–355]. Subunit phosphorylation is complex, and is mediated in part by PKA, PKC, c-jun N-terminal kinase (JNK) or Ca^2+^calmodulin-dependent protein kinase 2 (CamKII), and directly or indirectly by dephosphorylation by protein phosphatase 1 (PP1) or calcineurin/protein phosphatase 2B [356–358]. These events are usually initiated, either directly or indirectly, by the elevation of post-synaptic Ca^2+^ (Fig. 3). This is mediated either by NMDA Rs (most commonly in Hebbian, synapse-specific plasticity) or else by mGluRs or by voltage-dependent Ca^2+^ channels (VDCCs).

VDCCs are capable of mediating induction of LTP and LTD, for example L-type (Ca_v_1) channels can initiate HF LTP [359–361], and a mutant gain-of-function form of*Cacna1c* (Ca_v_1.2) produces enhanced late LTP in mouse HF [362]. In the atypical case of mossy fibre-CA3 synapse HF LTP, R-type Ca_v_2.3 channels appear to be involved [363]. LTP at corticothalamic synapses onto relay neurones is NMDA R-dependent, whereas LTD is dependent on L-type Ca^2+^channels [364]. Other contributions of voltage-dependent Ca^2+^ channels to plasticity exist—for example LTD of communication between TRN neurons is mediated by T-type (Ca_v_3) channels [365], and T-type channels are also required for LTP of corticothalamic and thalamocortical collateral input onto TRN neurons [297, 366, 367].

Apart from mediating AMPA R phosphorylation, kinase cascades also operate to orchestrate actin-regulatory proteins and mediate spine enlargement or retraction in LTP or LTP respectively [368] (Fig.3). The state of activation of these kinase signalling cascades, and the small GTPases at their head, are governed by a diverse family of GTPase regulatory proteins, which when triggered, lead to activation (via guanine nucleotide exchange factors—“GEFs”) or inhibition (via GTPase activating proteins—“GAPs”, or guanine nucleotide dissociation inhibitors—“GDIs”) of the GTPase. The Ras pathway is well-characterised for its role in LTP induction [369]. The Ras-Raf-MEK-ERK cascade is linked to the long-term maintenance of LTP via transcriptional actions (some of which are mediated via CREB phosphorylation). In addition, ERK activation also produces more rapid effects on synaptic plasticity [370–372]. Pharmacological inhibition of ERK blocks LTP, and may also interfere with mGlu R—induced LTD [373, 374]. Activating mutations of KRas, or functional deletion of HRas, or of the RasGEFs RasGRF1/2, or of the RasGAP neurofibromin type 1, produces LTP impairment in mice [375–377] and intellectual disability in humans [378]. Thus either activation or inhibition of this pathway causes plasticity impairment. It is worth noting that the mutations in the genes involved in these core pathways tend to result in intellectual disability and cognitive impairment, but not schizophrenia [378].

LTP and LTD in PFC and HF are profoundly influenced by dopamine receptor stimulation [95, 98, 379]; D2 antagonists act to suppress PFC and HF plasticity while both D1 and D2 agonists tend to promote plasticity (LTP and LTD) and suppress LTP reversal (depotentiation) [6, 161–163, 380]. The dopaminergic influence is probably via modulation of PKA [380–382] or intracellular Ca^2+^levels [383].

### Genetic Evidence that Dysfunctional Cortical Synaptic Plasticity Contributes to the Causes of Schizophrenia Overview

There is a strong genetic contribution to the causes of schizophrenia. Cumulative burden from common sequence variants at more than 200 genetic loci is implicated [3], each variant individually of small effect. There are also rare CNVs which, on their own, substantially increase schizophrenia risk [147, 384–388].

- The Primary Molecular Mediators of Homosynaptic Plasticity
  There are groups of functionally related genes among the hundreds of genomic loci identified by genome-wide association studies (GWAS) of genetic variants that are common in the population, suggesting some commonality or convergence of mechanism for the numerous genes. These strongly implicate synaptic glutamate signalling via NMDA Rs [3, 295–298, 367]. Patients with schizophrenia show an increased burden of common variants (or indeed occasionally rare mutations, or copy number variants (CNVs—a deletion or duplication of a chromosomal region)), in genes related to NMDA R signalling complexes, and to Arc-interacting proteins (although not specifically the *ARC *gene itself) [3, 389–395].
  i. Glutamate Receptors
  Mutations in *GRIN2A*, the gene encoding the GluN2A NMDA R subunit, have received a great deal of attention recently, once it became clear that there are not only common variants of small effect increasing schizophrenia risk in the general population, but also rare variants that massively increase risk on their own [3, 396]. In fact, many of the genes encoding ionotropic glutamate receptor subunits have genetic variants (both rare, large effect and common, small effect) associated with schizophrenia risk (summarised in Table1). GluN1 and GluN2B variants are notable by their absence. GluN1 mutations do occur, but are associated with a syndrome of epilepsy with severe developmental delay and intellectual disability [408, 409]. Similarly, GluN2B mutations tend to cause developmental delay [410], although there are differing symptom profiles associated with loss-of-function versus gain-of-function variants. The *GRIN2A *mutations linked to schizophrenia appear to be mainly loss-of-function variants [399], consistent with the concept that hypofunction at NMDA Rs contributes to the aetiology of the condition. Considering the prominent role of NMDA Rs in synaptic plasticity, this is strong support for the concept that impaired plasticity mechanisms lie at the root of neural circuit dysfunction in the condition.

**Table 1:** LTP inducers and risk for schizophrenia. Gain/Loss indicates where it is possible to predict whether the genetic influence leads to a gain or loss of function, based on the copy number or the nature of the rare mutation

Gene	Protein	Complex	Common?	Rare?	Gain/Loss?	Citations
*GRIN1*	GluN1	NMDA R				
*GRIN2A*	GluN2A	NMDA R	yes	yes	Loss	[3, 389, 396–400]
*GRIN2B*	GluN2B	NMDA R				
*GRIN2C*	GluN2C	NMDA R		yes	Loss	[398, 401]
*GRIN2D*	GluN2D	NMDA R		yes	Loss	[401]
*GRIN3A*	GluN3A	NMDA R		yes	Loss	[398, 400]
*GRIN3B*	GluN3B	NMDA R				
*GRIA1*	GluA1	AMPA R				
*GRIA2*	GluA2	AMPA R				
*GRIA3*	GluA3	AMPA R		yes		[396]
*GRIA4*	GluA4	AMPA R				
*GRIK1*	GluK1	Kainate R				
*GRIK2*	GluK2	Kainate R		yes		[402]
*GRIK3*	GluK3	Kainate R		yes		[396]
*GRIK4*	GluK4	Kainate R	yes			[403]
*GRIK5*	GluK5	Kainate R		yes		[404]
*GRM1*	mGlu1	mGlu R (Gp1)	yes			[3, 398]
*GRM3*	mGlu3	mGlu R (Gp2)	yes			[3]
*GRM5*	mGlu5	mGlu R (Gp1)		yes		[405]
*GRM7*	mGlu7	mGlu R (Gp3)		yes		[398]
*CACNA1S*	Ca_v_1.1	L-type	yes	yes		[3, 268, 269, 389, 394]
*CACNA1C*	Ca_v_1.2	L-type	yes	yes	Loss	[3, 268, 269, 394, 406]
*CACNA1D*	Ca_v_1.3	L-type	yes	yes		[3, 268, 269]
*CACNA1F*	Ca_v_1.4	L-type				
*CACNA1G*	Ca_v_3.1	T-type	yes	yes		[396, 406]
*CACNA1H*	Ca_v_3.2	T-type		yes	Loss	[394]
*CACNA1I*	Ca_v_3.3	T-type	yes	yes	Loss?	[3, 230, 268, 269, 406, 407]
*CACNA1A*	Ca_v_2.1	P/Q-type				
*CACNA1B*	Ca_v_2.2	N-type		yes	Gain, Loss	[394, 402]
*CACNA1E*	Ca_v_2.3	R-type	yes			[406]
*CACNA2D1*	Ca_v_a2d-1			yes	Loss	[394]
*CACNA2D2*	Ca_v_a2d-2			yes	Loss	[3, 394]
*CACNA2D3*	Ca_v_a2d-3					
*CACNA2D4*	Ca_v_a2d-4			yes	Loss	[394]
*CACNB1*	Ca_v_b2					
*CACNB2*	Ca_v_b2		yes	yes	Loss	[394, 406]
*CACNB3*	Ca_v_b2					
*CACNB4*	Ca_v_b4			yes	Loss	[394]
  The *GRIA3* gene, encoding the GluA3 AMPA R subunit, is similar to the *GRIN2A *gene, in that rare genetic variants are among those individually conferring the greatest risk for schizophrenia [396], although as yet there is no reported association for common variants. The metabotropic glutamate receptor genes GRM1, 2 and 3 are all located at schizophrenia risk loci identified by GWAS (GRM3 most convincingly), as is the gene encoding the D2 dopamine receptor [3].
  ii. Voltage-Sensitive Ca2+ ion Channels
  The genetic evidence supporting VDCC dysfunction as contributing to schizophrenia aetiology is also very strong (Table 1). It covers most subunits and channel subtypes. The evidence is particularly robust for L-type and T-type channels, which have the clearest roles in LTP (Table 1). These seem to be mainly loss-of-function. Consistent with the overt role of T-type channels in thalamic plasticity, a schizophrenia-associated rare *Cacna1i* variant (Ca_v_3.3) disrupts TRN function in mice [411]. The lack of evidence that variants in genes encoding the Ca_v_1.4 channel or P/Q-type channels contribute to disease risk is noteworthy (Table 1). The Ca_v_1.4 channel is not expressed in the brain, while P/Q-type channels are largely presynaptic and mediate Ca^2+^-dependent exocytosis, along with paired-pulse facilitation. This may suggest that this particular form of plasticity is not compromised in schizophrenia.
  Overall, the weight of evidence summarised in Table 1 would strongly suggest that plasticity processes are likely to be compromised in schizophrenia.
- The Secondary Molecular Mediators of of Homosynaptic Plasticity
  It should be noted that the following account of the mediators of synaptic plasticity is inevitably simplified to some extent. Studies investigating the role of particular molecules in synaptic plasticity are numerous, and not all the evidence that they report is consistent. Indeed, some of it is mutually exclusive. Studies vary in their strengths and weaknesses: issues such as inadequate statistical power (increasing risk of false-negative findings), and the use of genetically modified mice on mixed genetic backgrounds (increasing risk of both false-positive and false-negative findings), occur repeatedly. The following sections attempt to pull out the strands of consistent and reliable information, and weave these into a representative yet clarified depiction of the evidence. Molecules that only affect synaptic plasticity indirectly (for example by hindering neurotransmitter release or post-synaptic synaptic signal transduction, or by distorting synapse architecture) are not usually mentioned, as almost any molecule that contributes to the structure and function of presynaptic terminals or dendritic spines and their signalling roles will affect plasticity as part of a wider impact on neurotransmission. Here, I focus on the core signalling components—genes/proteins whose major function at synapses is in relation to plasticity.
  The neurochemical mechanisms involved in the best-characterised forms of synaptic plasticity (LTP and LTD), (mainly studied in the hippocampus) are shown in Fig. 3. The kinases CamKIIα, PKC, PKA and ERK are central players in LTP [347], as are the phosphatases calcineurin and PP1 in LTD [8, 412, 413]. Scaffold proteins are likely to be important for compartmentalising the signalling [414, 415].
  The regulation of synaptic AMPA R number is a key component of synaptic plasticity, with AMPA Rs cycling between synaptic, extra-synaptic and intracellular locations. The initial phase of activity-dependent AMPA R internalisation involves dynamin 3 (DNM3), and long-term disruption of DNM3 function leads to a loss of synaptic AMPA Rs [416] and compromised LTP [417]. Rare variants in the *DNM3 *gene dramatically increase schizophrenia risk [396].
  Rab GTPase proteins are coordinators of membrane vesicle recycling [418]. The endocytosis of AMPA Rs involves the action of Rab5 [152, 419–421], while exocytosis involves Rab11 [422–424], 4.1R/*EPB41*[425], Rab39B [426] and Syntaxin 4 (*STX4*) [427, 428] (Figs.3 and 4). These are likely to be the ultimate mediators of AMPA R turnover at the synapse. Indeed, *Stx4* or *Rab39b *disruption impairs HF LTP [428, 429]. *EPB41 *lies at a genomic locus associated with schizophrenia risk by GWAS [3], a rare mutation has also been identified in a proband [389], and a rare missense variant in the *STX4 *gene has been identified in probands but not in controls [398]. Microduplications containing the *RAB39B *gene also increase schizophrenia risk [430].

**Fig. 4:**
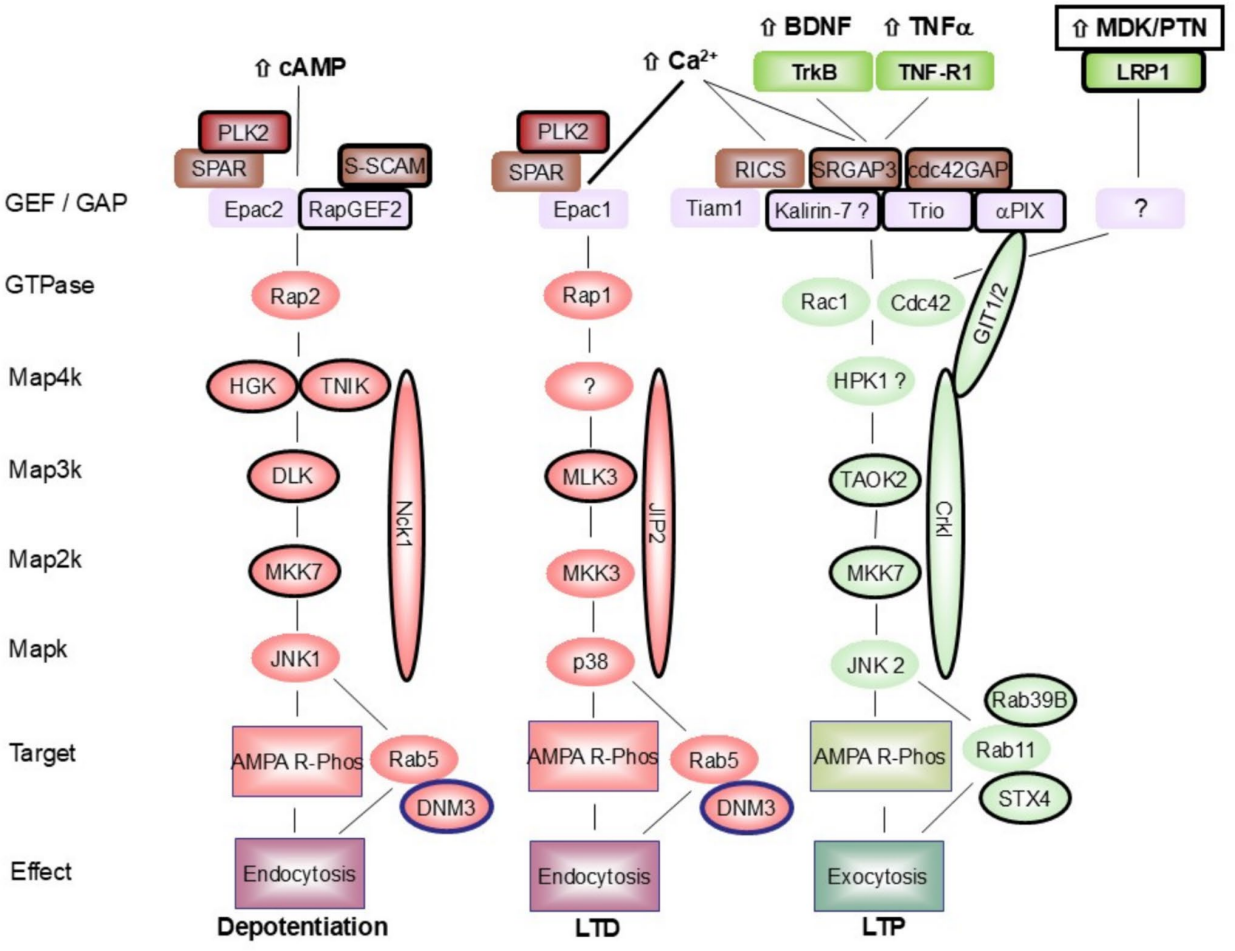
Homosynaptic plasticity: secondary modulators. Bold outline for a symbol indicates that genetic evidence suggests a contribution to schizophrenia aetiology
  The retromer complex is another important regulator of endocytosis in many cell types, and among its roles is the internalization of AMPA Rs during LTP [418, 431, 432]. The genes encoding two components of the retromer complex, Vps29 and Tbc1d15, have both been associated with schizophrenia risk by GWAS [3].
  Of note, where the LTP is due to synaptic incorporation of kainate Rs rather than AMPA Rs, Rab17 appears to be involved in the exocytosis rather than Rab11 [433]. Consistent with the impression from Table1that plasticity of kainate Rs may also be implicated in schizophrenia aetiology, a rare missense mutation in Rab17 has been identified in patients but not controls [398].
  i. Scaffold Protein
    These distinct signalling cascades are held together as functional units by scaffolding proteins, which play an essential role in coordinating and separating distinct modules. Kinase suppressor of Ras (KSR) acts as a scaffold protein for Ras-Raf-MEK-ERK signalling, and plays an important role in LTP [414] (Fig.3). The Shank and DLG (e.g. PSD-95) families of post-synaptic scaffolding proteins are clearly associated with schizophrenia risk through variants, both common and rare, in a number of gene family members [434]. Shank3 in particular is detected at both a GWAS-significant locus [3] and in a screen for rare, high-penetrance variants [396], although for both Shank and DLG families, the interpretation is clouded slightly by the fact that variants can affect general synaptic structure and function, rather than being specific for plasticity processes. Shank3 is a substrate for protein phosphatase 1 (PP1) [415], and both PP1 and Shank3 are necessary for synaptic AMPA R localization and the full expression of LTP [413, 435]. The gene encoding Nck1 is at a locus of GWAS-significant risk for schizophrenia [3], with rare genetic variants also increasing schizophrenia risk [396]. Nck1 interacts with the eponymous TNIK (Traf2 and Nck1-interacting kinase) downstream of TNF∝ signalling via TNF-R1-TRAF, and also with Hepatocyte Progenitor Kinase-Like/Germinal Center Kinase-Like Kinase/Nck1-interacting kinase (HGK, NIK, *MAP4K4*), which is a Rap2 effector [436] (Fig.4). The genetic data implicating TNIK and MAP4K4 in schizophrenia is quite strong (see below).
    The *Mapk8ip2*/islet brain 2/c-jun N-terminal kinase-interacting protein 2/JIP2 gene is at a GWAS-significant locus [3], and is also one of a number of genes where a missense variant may increase the penetrance of the 22q11 deletion that markedly elevates schizophrenia risk [437]. It transduces aspects of NMDA R signalling [438].
    There is a direct interaction between Crkl (the *Crkl *gene being located within the high-penetrance 22q11 deletion locus) and both JNK2 [439] and hematopoietic progenitor kinase 1 (HPK1, *MAP4K1*) [440], mentioned below.
  ii. GTPases
    The super-family of low molecular-weight GTPases is very large. Members of the Rab family, involved in endo-and exocytosis, have been considered already, in terms of AMPA R recycling. However, the proteins in many other branches of the superfamily play important roles in neuronal signalling in general, and in synaptic plasticity in particular. Of the different isoforms of Ras, *NRAS *resides at a locus associated with schizophrenia risk by GWAS [3], but does not seem to have been studied specifically in terms of a role in synaptic plasticity. Blockade of Ras actions in general prevents synaptic delivery of AMPA Rs and blocks LTP [441]. For Ras regulatory proteins, while there is no overt evidence associating *RASGRF1 *variants with schizophrenia risk, there is a possible link to RasGRF2 via a duplication segregating with familial schizophrenia [397]. Mice lacking RasGRF2 show greatly impaired LTP in HF [442], whereas genetic deletion of RasGRF1 impairs LTD [443].
    GTPases of the Rho family (prototypically Rac, Rho and cdc42 proteins) are best known for their role as actin cytoskeleton organisers. For the alterations in spine morphology that represent an important aspect of synaptic plasticity, Rac1 and RhoB appear to be primarily involved [444, 445], with pharmacological or suppression of either gene impairing LTP [368, 446–448]. However, Rac1 inhibition or genetic deletion also impairs LTD [449], as does deletion of the Rac1 activator *Prex1 *[450]. The precise mechanisms linking Rac to either LTP or LTD are not fully defined, but Rac can activate a number of downstream kinases (e.g. protein kinase D (PKD), protein kinase N/protein kinase C-related kinase 1 (PKN/PRK1), p21-activated kinase 1 (PAK1), discussed in the following section). Equally, another member of the Rho family, RhoG, can also be activated by Prex1 and promote endocytosis via Rab5 [451, 452].
    Certainly, disturbance of the function of any of the other components of the spine actin regulatory system—PAK1, RhoKinase/ROCK, Lim Kinase (LimK) and cofilin—impairs LTP [446, 453, 454]. The dynamics of dendritic spine morphology are inextricably linked with synaptic efficiency, however, and the situation becomes more difficult to comprehend in full because these Rho GTPases also trigger other signalling cascades apart from those inducing cytoskeletal reorganization. The complex regulation of Rac signalling is also of interest, as will be discussed shortly, as there are other signalling modules subserving aspects of synaptic plasticity. These pathways mediating spine morphology flexibility are also implicated in vesicle fusion with the plasma membrane. Thus both PAK1 and RhoB are associated with recycling endosomes, and likely contribute to AMPA R delivery to synapses during LTP [455–457].
    For Rap and Rho (Rac family, Rho family and cdc42) GTPases, there is little evidence that genetic variants in the genes themselves increase schizophrenia risk. However, as with the Ras pathway, the regulatory proteins are clearly playing an important role. Kalirin is a Rac/Rho GEF, activated via phosphorylation by CamKII [458]. Increased *Kalirin *expression enhances AMPA currents [459]. Genetic deletion of an isoform of *Kalirin *in mice does not affect basal glutamatergic transmission [460, 461], although basal AMPA currents may be suppressed in immature neurons [458], but clearly impairs LTP, although LTD may also be reduced [458, 461, 462]. Kalirin, via Rac1, is also a known interactor of Rab11 to regulate exocytosis [463]. This does not demonstrate, but is consistent with, a role for kalirin in the pathway from NMDA Rs or TNF-R1 to LTP promotion via JNK2 (Figs.3 and 4). It is supported by evidence that genetic deletion of *Rac1 *also impairs LTP without affecting basal transmission [447], and that Rac1 overexpression enhances AMPA R clustering and synaptic responses to glutamate [464]. Similarly, genetic deletion of cdc42 also impairs LTP [465]. Both common, small effect and rare, large effect *KALIRIN *genetic variants are associated with schizophrenia risk [466–468].
    Trio (ARHGEF23) is a GEF for Rac and Rho family GTPases. As with *Kalirin*, over-expression of *Trio *enhances AMPA R signalling [459]. Haploinsufficiency or knockdown of Trio leads to reduced AMPA currents [469] and suppressed HF LTP [459, 470]. Trio is also genetically associated with schizophrenia, via rare missense mutations [396, 471]. Tiam1, which can link NMDA R stimulation to Rac activation [472], is also activated by CamKII phosphorylation [473], and this is essential for the expression of LTP [474]. Tiam2 is less well-characterised, but is very highly homologous to Tiam1, and a significant association signal for *Tiam2 *was detected in a focused schizophrenia GWAS and in an exome sequencing study [392, 398, 475]. Interestingly, the expression of Tiam2 is remarkably restricted to forebrain glutamatergic neurons [476].
    PAK-Interacting Exchange Factor Alpha (aPIX/*ARHGEF6*) acts as a GEF for Rac and Cdc42 proteins, and interacts with Crkl [477]. The *CRKL* gene lies at the 22q11 high penetrance schizophrenia locus. Further, while variants in the αPIX/*ARHGEF6 *gene itself do not appear to be associated with schizophrenia risk, αPIX forms a complex with either G-protein-coupled receptor kinase-interacting protein 1 (GIT1) or GIT2 (multi-functional scaffold proteins with GAP activity) to activate Cdc42 [478]. GIT1 is linked to the regulation of synaptic AMPA Rs [479], and rare missense variants in *GIT1 *with reduced function have been associated with schizophrenia in a number of cases [389, 394, 480], while common variants in *GIT2 *are associated with schizophrenia by GWAS [3]. Thus the evidence for compromised function of a CRKL/αPIX/GIT complex contributing to disease aetiology is strong. Further, mice lacking αPIX show intact basal glutamatergic transmission but reduced LTP and enhanced LTD [481], potentially providing a direct link between reductions in synaptic efficiency and disease risk.
    Asef1/ARHGEF4 has unclear specificity in regulation of cdc42/Rac/Rho GTPases [482, 483], but appears to promote AMPA R internalization [484], and rare variants are associated with elevated schizophrenia risk [396].
    RapGEF2 (PDZ-GEF1) activates Rap1 and Rap2 [485]. The gene lies within a microduplication segregating with schizophrenia in a family pedigree [486], and a rare missense variant has been identified in people with schizophrenia [398]. Mice lacking RapGEF2 have normal basal transmission but appear to have diminished LTP in HF (Jiang et al.2022, despite the molecular evidence better suiting a role for RapGEF2 in depotentiation (Fig. 2). A potential explanation is that a failure of depotentiation throughout development is predicted to result in a greater proportion of potentiated synapses at baseline, and a reduced ability to demonstrate further LTP.
    Epac2/*RAPGEF4 *is a cAMP-dependent Rap-GEF that can mediate a depression of synaptic efficiency [487], involving depotentiation and AMPA R internalization via Rap2 [488]. No overt evidence supports an association with genetic risk. However, the synaptic scaffolding molecule/membrane-associated guanylate kinase inverted-2 (S-SCAM/MAGI-2) protein binds to RapGEF2 [489], activates Rap1/2 [490], and promotes endocytosis [491] (Fig.2). Genetic deletion of S-SCAM/MAGI-2 results in a loss of synaptic AMPA Rs and reduced dendritic spine density [492], while over-expression increases synaptic AMPA Rs and impairs LTP [493]. Rare *MAGI2 *genetic variants dramatically increase schizophrenia risk [396].
    The Rho-GAP “RhoGAP interacting with CIP4 Homologs Protein 2” (Rich2/*ARHGAP44*) gene lies at a GWAS locus associated with schizophrenia risk [3]. Rich2 reportedly increases synaptic efficiency at glutamatergic synapses, promoting spine enlargement and AMPA R exocytosis in the same pathway by suppressing Rac1 and via an interaction with the post-synaptic scaffold protein Shank3 [494–496].
    RhoGAP involved In Beta-Catenin-*N*-Cadherin and NMDA Receptor Signalling (RICS/GRIT/*ARHGAP32*) is a Rac1/cdc42 GAP that interacts with Crkl scaffolds [497] and NMDA Rs [498, 499] and is inhibited by CamKII, hence mediating (in)activity-dependent suppression of this cascade. RICS mediates NMDA R-triggered exocytosis of GABA_A_Rs, and so possibly HSPlas up-scaling of inhibitory transmission [500]. This gene also may show association with schizophrenia risk [501].
    RhoB inactivators are also implicated in disease risk, in that a rare deletion of the *ARHGAP11B *gene has been identified in twins with schizophrenia-type diagnoses [502], also being present within the 15q13 microdeletion that increases schizophrenia risk [147], while a rare de novo missense variant in the closely-related *ARHGAP11A *gene was identified in a proband [503].
    Cdc42GAP/*ARHGAP1 *also lies at a GWAS locus associated with schizophrenia risk [3]. It does not appeared to have been tested for a possible role in synaptic plasticity, although it is clearly expressed in brain tissue according to various high-throughput databases, and preferentially in glutamatergic neurons [504]. SrGAP3/*ARHGAP14 *is a gene where microduplications have been associated with schizophrenia risk in a case–control study [505] and a potentially inactivating microduplication has also been found to segregate with disease in the family of a proband with childhood onset schizophrenia [506]. Altered dendritic spine morphology and cognitive behavioural deficits in mice lacking functional *Srgap3 *[507] suggest a likely role in synaptic plasticity, but this does not seem to have been directly tested yet.
    The RapGAP SPAR/*SIPA1L1 *acts to suppress activity of Rap1 and Rap2, but is rapidly degraded when phosphorylated by the polo-like kinase PLK2 [508]. This action of PLK2 is essential for down-scaling in HSPlas [508, 509]. Strikingly, the *PLK2 *gene is the sole gene at one of the GWAS loci associated with schizophrenia risk [3].
    Overall, the genetic evidence for disruption of Rac/Rho/Rap GTPase regulation in schizophrenia is striking, potentially impacting a range of downstream signalling pathways and their resulting contributions to the varied aspects of synaptic plasticity.
  iii. Kinases
    MAP kinases (ERK1, ERK2) are prominent mediators of LTP and LTD [369, 370, 374]. Erks are involved in promoting transcriptional alterations underlying late-phase LTP via phosphorylation of transcription factors such as CREB, but Erk activation also promotes Rab4/8 activity [510–512] (Fig.1). The ERK1/*MAPK3*gene lies within the 16p11.2 microduplication region associated with dramatically increased risk of schizophrenia [147, 385, 386, 391, 392] and this region also shows as contributing to risk via a common variant in GWAS [3].
    Many other members of the kinase superfamily are involved in plasticity mechanisms. CamKII and PKA have very well-characterised roles in LTP [513, 514]. PKA functions as a tetramer of two catalytic and two regulatory subunits, and the gene encoding one of the regulatory subunits, *PRKAR2A*, lies at a schizophrenia GWAS locus [3]. Deletion of this gene in mice impairs cortical LTP [515]. Rare *CAMK2A *mutations have been identified in patient cohorts [516].
    As noted earlier, Rac controls a number of kinase activities, and is implicated in both LTP and LTD. Protein kinase D (PKD) is a less well-characterised kinase family within the CAMK group. There are 3 genes (*PRKD1-3*), and remarkably, two of them (*PRKD1* and* 3*) are found at loci significant in schizophrenia GWAS [3]. PKD is activated downstream of Rac [517], and its activation contributes to activity-dependent AMPA R endocytosis [518] (Fig.3). This LTD pathway probably also involves another Rac1 effector PKN1/PRK1, since genetic deletion of *Pkn1 *increases synaptic AMPA Rs [519].
    Hematopoietic Progenitor Kinase 1 (HPK1, *MAP4K1*) is very closely related to Hepatocyte Progenitor Kinase-Like/Germinal Center Kinase-Like Kinase/Nck1 interacting kinase (HGK/NIK/*MAP4K4*), and more distantly related to two other members of the same group—another Nck-interacting kinase TRAF2 and NCK Interacting Kinase (TNIK), and thousand and one kinase 2 (TAOK2). The *TAOK2 *gene is located within the 16p11.2 microduplication region associated with dramatically increased risk of schizophrenia [392] and also implicated in the common disease via GWAS [3], and via rare mutations as well [396]. A rare de novo missense variant in HGK/NIK/*MAP4K4 *was identified in a proband [503], and a rare missense *TNIK *variant is present in all affected members of a family with schizophrenia over four generations [520]. Common *TNIK *variants have also been associated with the degree of PFC hypofunction in patients [19, 26]. Since Rap2-TNIK-JNK signalling has been linked to AMPA R endocytosis and depotentiation [521], this signalling module is highly likely to link NMDA Rs to depotentiation. In contrast, Hpk1 interacts with the exocytotic machinery mediating synaptic delivery of AMPA Rs [428, 522], while loss of Taok2 compromises synaptic AMPA R localisation and measures of synaptic plasticity including *Egr1 *induction [523, 524].
    The JIP2 module also includes MLK3/*Map3k11*, encoded by a gene at a schizophrenia GWAS-significant locus [3, 525]. JIP2 tethers MLK3 and p38 kinase into a module with Tiam1/2 and MKK3 [525] in the post-synaptic density [526] (Fig.2). MLK3 can be activated by various Map4ks including HGK and HPK1.
    Basal glutamatergic transmission is elevated, while LTP is not impaired, in DLK (*Map3k12*) knockout mice [527]. An equivalent elevation of basal transmission but lack of effect on LTP is also seen with pharmacological inhibition of JNK [528]. DLK is present in the PSD, and lies between TNFα and JNK in a signalling module leading to JNK activation [527, 529]. The DLK/*MAP3K12 *gene also lies at a genomic locus associated with schizophrenia risk by GWAS [3], and a rare missense variant has been reported in a schizophrenia proband [398].
    The actions of p38 seem nearly always to involve decreases in synaptic efficiency. p38 mediates LTD, but not LTP, induction in HF CA1 region [530–532]. p38 suppresses HF DG LTP [533], and p38 also reportedly mediates suppression of LTP and internalisation of GluA1-containing (and not GluA2-containing) AMPA Rs in HF CA3 [534]. LTD in HF DG is dependent on p38 and L-type Ca^2+^channels, and, perhaps surprisingly, as discussed below, on TNF-R1 [535, 536]. Equivalent effects have been observed in cerebral cortex [537]. Synaptic GluA2-containing AMPA Rs are removed by p38 activation (downstream of Rap1) [521]. Hence, although there are 4 different genes encoding p38 isoforms, there is a general picture where p38 activation acts to reduce synaptic efficiency. None of the p38 genes shows obvious association with schizophrenia risk.
    Activation of ERK, JNK, p38 and L-type Ca^2+^channels is required for the expression of LTP in anterior cingulate cortex [538]. There are 3 JNK isoforms, activated by the upstream kinases MKK4 or MKK7. JNK1, JNK3 and MKK7, but not JNK2 or MKK4, are present in the post-synaptic density [358], and the JNK modules seem to exert dichotomous actions in plasticity. Broad pharmacological inhibition of JNK (JNK1-3) suppresses LTD in HF dentate and CA1 [533, 539, 540], without affecting LTP [528, 533, 539], or else enhances LTD while suppressing depotentiation of LTP in dentate [541]. The latter finding is supported by evidence that depotentiation of LTP is mediated by Rap2-TNIK-JNK(1–3)-triggered internalisation of synaptic GluA1/2-containing AMPA Rs [521, 542] (Fig.2). There is also evidence for a non-synapse-specific but rapid up-scaling of synaptic function via JNK [543]. Indeed, JNK1 promotes synaptic insertion of GluA2/4-containing AMPA Rs [358], and JNK1/2 (not JNK3) hinders AMPA R internalisation [544]. Genetic manipulations allow a more precise examination of the roles of JNK isoforms in plasticity. JNK1 KO mice have impaired HF LTD [545], while JNK2 KO mice have impaired hippocampal NMDA R-dependent LTP [546]. The apparently contradictory or bi-directional findings may be largely resolved if JNK1 tends to promote depression/depotentiation and JNK2 promotes potentiation.
    JNK isoforms 1–3 are activated by upstream kinases MKK4 (*MAP2K4* gene) and MKK7 (*MAP2K7* gene). *MAP2K7 *is both reduced in expression in PFC from patients with schizophrenia, and genetically associated with risk for schizophrenia with a large effect size for a common variant [547, 548]. Rare variants of the *MAPK9 *(JNK2) gene were found more commonly in patients than in control subjects [396], splice variants of the *MAPK9 *(JNK2) gene were also identified in an exome sequencing study for variants potentially increasing penetrance of the 22q11 deletion [437], a focussed study reported association of *MAPK10 *(JNK3) variants with disease risk [549], and rare missense mutations or duplications involving the *MAPK8 *(JNK1) gene have been identified in some patients [389, 550]. Hence there is substantial evidence implicating JNK signalling in disease aetiology. This is supported by evidence that mice hemizygous for a deletion of the gene encoding MKK7 show a range of schizophrenia relevant phenotypes including metabolic hypofrontality and compromised sensorimotor gating, with impaired working memory, attention and cognitive flexibility [548, 551–553].
    Phosphatases act to dephosphorylate target proteins and oppose the actions of kinases. The phosphatase PP1 is activated during LTD and inhibited during LTP, while PP1 inhibition can block LTD [413]. Common, small effect, variants in four different PP1 catalytic or regulatory subunit genes also associated with schizophrenia risk [3], so this module could potentially play quite a prominent role in disease aetiology. Equally, a rare variant in a calcineurin subunit gene is found at higher frequency in patients than in control subjects [396].
- The Molecular Mediators of Late-Stage Plasticity
  The late phase of plasticity is sustained by altered gene transcription, and morphological or even numerical changes in dendritic spines.
  As noted above, *Egr1 *is one of the best-characterised molecular markers for synaptic plasticity induction in cortex and HF [180, 181, 254, 273, 276, 278, 279]. EGR1 plays a critical role in the long-term plasticity responses [554], via secondary transcriptional regulation of a number of genes involved in synapse function and the control of synaptic protein turnover [555, 556], potentially including *ARC *itself [557]. There is evidence that compromised EGR1 function may also contribute to schizophrenia risk, in that the *EGR1 *gene lies at a schizophrenia GWAS-significant locus [3], and, as noted earlier, *EGR1 *expression is robustly decreased in PFC from patients with schizophrenia [283–286], although expression is reportedly increased in auditory cortex [316].
- The Primary Molecular Mediators of Homeostatic Plasticity
  i. TNFα
    Interestingly, these less prominent, plasticity-modulating pathways also act to mediate cytokine influences on synaptic function. Of these cytokine effects, those of TNFα have received particular attention, as they are believed to have network-level effects as well as synapse-specific effects, although there are areas of controversy. LTD in HF DG is dependent on p38 and on TNF-R1 [535, 536]. Hippocampal LTD is compromised in mice lacking TNFα [558], and physiological concentrations of TNFα rapidly suppress hippocampal (CA1/DG) LTP via p38 and JNK [559–561], although enhancing effects on LTP are also reported [562, 563]. Physiological concentrations of TNFα also reportedly increase synaptic GluA2-containing AMPA Rs via TNF-R1 and p38 [564, 565], and, at higher concentrations, TNFα-induced HSPlas increases in synaptic GluA2-lacking AMPA Rs occur alongside internalisation of GABA_A_Rs, also via p38 [303, 566, 567]. In this paradigm, sustained inactivity triggers increased sensitivity to excitation—HSPlas up-scaling. Indeed, TNFα expression is elevated in cultured neurons by activity suppression using TTX [568]. [569]. This involves peri-synaptic astrocytes or microglia, which sense the drop in synaptic glutamate and release TNFα [303, 569]. This is interesting considering that evidence implicates astrocytic but not neuronal p38a in CA1 LTD [570]. HSPlas up-scaling in cortex is also reported to be TNFα-mediated [571].
    But how could TNFα both enhance (as in HSPlas up-scaling) and suppress (as in depotentiation and LTD) synaptic efficiency? This must reflect the ability of TNFα to recruit distinct signalling modules in different cellular contexts. TNFα can activate the RapGEF2-Rap2-TNIK/HGK-DLK cascade [529, 572], with both HGK and TNIK capable of mediating JNK activation by TNFα in peripheral cells [573, 574]. At CNS synapses, this is predicted, although again not yet demonstrated, to result in receptor endocytosis, and this may be the mechanism whereby TNFα suppresses LTP. This action may be favoured where post-synaptic Ca^2+^ levels are elevated, with Ca^2+^-sensitive Epac1/2 (*RapGEF3/4*) activating Rap1/2 GTPases to stimulate p38 or JNK1 signalling and AMPA R endocytosis in LTD [575]. The TNFα–DLK–JNK signalling module likely leads to AMPA R endocytosis [527, 529]. Equally, since p38 can activate the GTPase Rab5, and there is consistent evidence that Rab5 activation enhances synaptic AMPA R internalization [152, 419, 420, 576], we can also propose a TNFα-p38–Rab1 cascade as operating to down-regulate synaptic efficiency.
    The mechanisms of TNFα-induced rapid upregulation of synaptic efficiency are less clear. It is conceivable that p38a is involved in release of TNFα during up-scaling. p38 activity can trigger shedding of active TNFα from the cell membrane [577, 578], and glutamatergic activation of astrocyte mGlu3 receptors suppresses p38 activity [579], so a reduction in glutamatergic signalling may lead to elevated astrocyte p38 activity, triggering release of TNFα. The released TNF∝ can then activate a Rac/cdc42-HPK1-JNK2 cascade, as demonstrated in immune cells [580, 581], with the predicted, but as yet not demonstrated, result of increased synaptic AMPA R delivery. Consistent with this, the Rab11-FIP5 protein, which associates with Rab11 to orchestrate synaptic delivery of AMPA Rs [582], is phosphorylated during up-scaling [583] (Figs.2 and 5).

**Fig. 5:**
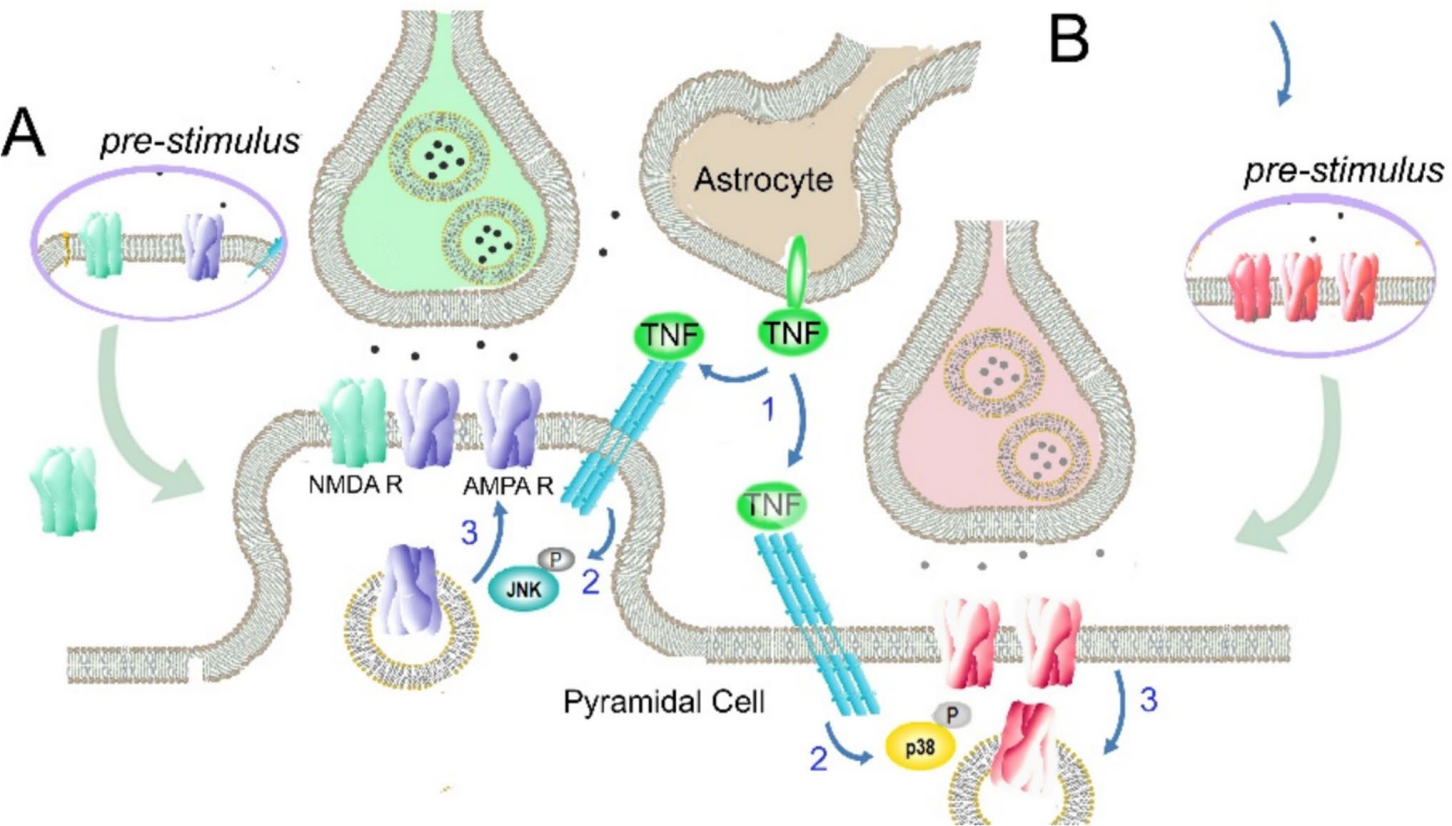
Up-scaling HSPlas mechanisms at (**A**) an excitatory synapse and (**B**) an inhibitory synapse, onto a glutamatergic neuron. Following suppressed levels of synaptic activity, TNFα is released from close to these synapses (1), activating p38 and JNK (2), and facilitating AMPA R exocytosis and GABA_A_ R endocytosis (3). The overall result is elevated AMPA R number and reduced GABA_A_ R number at these synapses
    There is some genetic evidence potentially linking TNFα-induced plasticity to schizophrenia. A rare missense mutation in the TNFα-induced protein 1 (*TNFAIP1*) gene was discovered in a patient with child-onset schizophrenia, [389], and increased frequency of rare mutations in people with schizophrenia relative to control subjects was also reported in another study [396]. The gene is activated transcriptionally by TNFα, and while the function of this gene in the brain is not well-characterised, the protein is a known interactor with RhoB and a regulator of JNK activity [584]. In fact, TNFα induces RhoB expression in peripheral cells, thereby activating JNK and p38 [585, 586]. A similar process in neurons would potentially contribute to HSPlas up-scaling.
  ii. Neurite Growth-Promoting Factor/Midkine/MDK and Pleiotrophin/PTN
    Recently, the immune system/CNS protein midkine/neurite growth-promoting factor (derived from the *MDK*gene) has been proposed as an additional primary mediator of HSPlas up-scaling [587, 588]. MDK and pleitotrophin (PTN) are the two members of a discrete cytokine family with neurite outgrowth-promoting activity, released during immune responses, but, as with TNFα, also playing a role in homeostatic plasticity. Both are relatively highly expressed in glutamatergic pyramidal neurons in cortex and HF [504]. LRP1 is one of their main receptors [589, 590]. Consistent with (at least partial) mediation of MDK effects, reduced cortical or hippocampal expression of either MDK or LRP1 reduces synaptic AMPAR levels and impairs plasticity [591, 592]; [587, 588]. While less well-characterised, the same appears to be the case for PTN [593], although high concentrations of PTN reportedly produce a dramatic suppression of HF LTP [594]. Genetic deletion of *Lrp1* also reduces *Arc *expression [595].
    Remarkably, *MDK, PTN* and *LRP1* are all at highly-significant GWAS loci [3], and rare mutations in *LRP1 *have also been found in people with schizophrenia on a number of occasions [511, 512, 596–599], so there is a sound basis for the hypothesis that genetic variants disrupting MDK/PTN-LRP1 mediation of homeostatic up-scaling could contribute to schizophrenia aetiology.
    LRP1 is complexed with NMDA Rs post-synaptically [600]. Stimulation of LRP1 activates Rac1 [601] (although the GEF/GAP responsible is unclear) and triggers Rab11-dependent exocytosis [602]. One of the additional consequences of LRP1 activation is a regulation of JNK signalling [603], so the pathway can be integrated with the plasticity mechanisms shown in Fig.4.
  iii. BDNF
    Brain-derived neurotrophic factor (BDNF) is released during high-frequency firing of glutamatergic fibres in cortex and HF, and its expression is also induced transcriptionally under these conditions [604, 605]. BDNF release is sufficient to induce LTP within a few minutes, via its receptor TrkB [606]. However, BDNF release also triggers HSPlas down-scaling over a longer time-period, due in part to induction of *Arc* and *Nptx2* gene expression [289, 290, 294]. CRKL (gene locus within the 22q11 deletion region) acts as a scaffold for TrkB signalling to modulate synaptic AMPA R number [607, 608].
    BDNF, released from glutamatergic cells in cerebral cortex and hippocampus, also acts via its receptor TrkB to upregulate components of GABAergic signalling (e.g. the GABA synthetic enzymes GAD65, GAD67 and Pvalb) [290, 609, 610]. When activity-dependent release of BDNF is restricted, this increases E/I balance inappropriately, by reducing *Pvalb* expression and Pvalb^+^cell activity [609, 611]. Haploinsufficiency of *Bdnf* in mice is sufficient to cause schizophrenia-like deficits in thalamocortical circuitry [612], γ oscillatory activity and cortical *Pvalb* expression [613]. TrkB deletion In Pvalb^+^cells leads to E/I imbalance in PFC [614]. Thus reduced BDNF function produces schizophrenia-like neurobiological changes. We recently discovered that the effect of BDNF on *Pvalb* and Gad1/2 expression is mediated by JNK signalling [320], as is the ability of BDNF to alleviate depression [615]. In fact, *Pvalb* expression is very powerfully regulated by JNK activity in cortical neurones.
    There is scant evidence that the *BDNF*gene [616], and none that the *NPTX2* gene, are genetically associated with schizophrenia, but BDNF expression is reduced dramatically in post-mortem PFC tissue from schizophrenia patients [617–619]. *Nptx2 *KO mice show elevated synaptic activity [304], consistent with impaired suppression of feedback or feed-forward inhibition. Glutamatergic synaptic activity is elevated in neuronal cultures from *Nptx2* KO mice (E18, 7 DIV) [289], suggesting that appropriate levels of GABAergic inhibition have not developed, even prior to *Pvalb* expression. The ability of BDNF to enhance activity observed in wild-type cultures is absent in cultures from *Nptx2* KO mice [289], as is the homeostatic down- or upmodulation of GluA4-containing AMPA Rs in Pvalb^+^ cells, by GABA_A_receptor blockade or TTX [304].
  iv. ARC
    Arc is an activity-dependent gene which regulates numbers of postsynaptic glutamatergic AMPA Rs [295–298, 367]. Arc expression is induced by NMDA R stimulation, or BDNF stimulation of TrkB, via ERK activation [620–624]. Haploinsufficiency of the *Arc* gene in mice conveniently models the neurobiological effects of the cumulative burden of many small-effect genetic variants in Arc-interacting genes. *Arc* KO mice do show hyperactivity of the mesostriatal dopamine pathway, and hypoactivity of the mesocortical dopamine pathway [625]—a reciprocal dysfunction which in humans is postulated to underly positive and negative/cognitive symptoms of schizophrenia respectively. Indeed, *Arc* seems to play an important role in the development of corticothalamic (CT) circuitry, since ubiquitous genetic deletion of *Arc* in mice suppresses hippocampal/PFC oscillatory activity [626]. Full deletion of the *Arc* gene compromises LTP—*Arc* KO mice have elevated AMPA R levels at hippocampal glut-glut cell synapses, enhanced early phase LTP, absent late-phase LTP and reduced LTD [306, 627].
    People with schizophrenia show an increased burden of rare mutations or CNVs in groups of functionally related genes, most prominently for the NMDA R signalling complex, and Arc-interacting proteins (although not specifically the *ARC* gene itself) [3, 389–391, 394, 395]. (The genes in these two groups overlap, but in each case the genetic association is not solely explained by over-lapping genes).
- The Molecular Maintainers of Synapse Structure
  As noted earlier, functional impairment of any of the many proteins involved in presynaptic or postsynaptic processes will adversely affect synaptic plasticity to some degree, but probably as part of a more general impairment in synaptic performance. Indeed, some genes with a very clear association with schizophrenia genetic risk are part of the framework that supports many aspects of excitatory synaptic function. These include neurexins, neuroligins and the DLG family of post-synaptic scaffolds [147, 391, 392, 628–632]. Their contribution to genetic risk for the disease is certainly consistent with some core dysfunction of plasticity processes.
  Disruption of any of the many hundreds of genes encoding proteins involved in the presynaptic processes of neurotransmitter release, the postsynaptic processes of receptor signalling or the presynaptic-postsynaptic integration and maintenance of synapses, will compromise synaptic plasticity to some degree, but alongside a more general impairment in synaptic function. The focus here is on evidence that synaptic plasticity, specifically, is affected in schizophrenia. However, one CNV that dramatically increases schizophrenia risk deserves a mention, as it contains just a single gene: a microdeletion at 2p16.3, containing a specific splice variant of the *Neurexin 1* gene (*NRXN1*α), which increases schizophrenia risk by ~ tenfold. Neurexins are presynaptic transmembrane proteins that bind postsynaptic partners to form and maintain synapses. The small deletions dramatically increasing schizophrenia risk affect *NRXN1*α, but not the shorter *NRXN1*β splice variants [633]. Rare, loss of function mutations in *NRXN1*α also markedly increase risk or potentially cause schizophrenia [396, 633, 634]. This clearly implicates diminished *NRXN1*a function in schizophrenia aetiology. Further, NRXN1α seems, like Arc, to regulate post-synaptic AMPA R endocytosis [635].

## Environmental Risk Factors in Relation to Synaptic Plasticity

Schizophrenia is caused by interaction of genetic and environmental (GxE) risk factors. The epidemiological evidence is fairly clear, that maternal infections in first and second trimester, but not third trimester, increase the risk of offspring suffering with schizophrenia decades later [636–639]. The elevated risk due to gestational severe maternal stress also operates in first and second trimester [4, 640–645]. Hence the initial direct effect of the gestational risk factor must affect the developing foetal CNS at that time—leading to a lasting detrimental influence. Thalamocortical synapses have formed by the end of the 1 st trimester, and astrocytes are also present in the cerebral cortex at this stage [646]. Hence the anatomical substrate is present for environmental influences in 1st or 2nd trimester to affect HSPlas at that time. The enduring effects of prenatal severe stress/infection are poorly understood, but, just as BDNF expression is reduced dramatically in post-mortem PFC tissue from adults with schizophrenia [617–619], expression of TNFα is elevated [647]. Although the elevated PFC TNFα levels have traditionally been viewed as a lingering marker of earlier immune activation, it should be remembered that elevated TNFα, and indeed suppressed BDNF, could also be evidence of an attempt to induce up-scaling of synaptic efficiency via HSPlas.

Few studies have addressed the effects of known environmental risk factors for schizophrenia on plasticity. In the absence of any element of genetic risk, prenatal immune activation in rats has little effect on HF LTP in offspring [648, 649], but may lead to a decrement in LTD in immature HF tissue [650]. Conversely, prenatal stress in rats reportedly produces an impairment in the expression of HF LTP in offspring while facilitating LTD [651, 652]. Stress in adulthood, as opposed to prenatally, seems to produce a clear decrement in LTP of various afferent inputs to the PFC, including hippocampal afferents [653], so there is at least the potential for mediators of stress, such as glucocorticoids, to impair plasticity.

Microglia, the brain’s resident immune cells, are present in the cortex from early in development, where they are understood to contribute to neural circuit formation and the pruning of unnecessary synapses [654, 655]. One of the strongest genetic associations with schizophrenia risk is in the region of the complement C4 genes [3, 656]. A popular hypothesis proposes that C4 overexpression resulting from the risk variant leads to excessive synaptic pruning by microglia during development, resulting in permanently reduced dendritic spine density [657–660]. A consequence of this may be deregulated structural plasticity. Inappropriate microglia activation may additionally act to compromise plasticity processes directly, as excessive TNFα release is predicted to cause unnecessary synaptic down-scaling [661]. Release of other cytokines also affects plasticity processes [539, 560] and microglial depletion in adulthood impairs LTP [662]. Thus microglial modulation of synaptic function and plasticity is likely to play a role in the cascade of events leading to the symptoms of schizophrenia.

It is important also to view the link between synaptic plasticity disruption and the development of schizophrenia symptoms through a developmental perspective. Mutations in NMDA R genes can cause developmental syndromes, illustrating the importance of NMDA R function for the formation and optimisation of neural circuitry [663]. NMDA Rs are clearly involved in the maturation of inhibitory interneurons, the setting of excitatory/inhibitory balance and the formation/maintenance of prefrontal circuitry [664–669].

As noted earlier, the *GRIN2A*gene, encoding the GluN2A subunit of the NMDA R, is especially interesting for understanding the actions of genetic risk, as it contains both common, small effect variants (hence contributing to the common condition) and also rare, very large effect variants [3, 396, 399]. CNS expression of the *GRIN2A *gene is well-documented as increasing substantially postnatally, rising dramatically in human from just prior to birth through to age 2–3 years, when levels are maintained through to adulthood [670–672]. Similarly in rodent, cortical *Grin2a *gene expression increases rapidly from ~ P7 (in mice) to adult levels [673–677].

This seems too late to be affected directly by prenatal maternal infection/stress. However, it is now clear that *GRIN2A *is also expressed in human (and mouse) cortex, at both mRNA and protein level, in first and second trimester [678–682], with levels possibly higher than those observed in the adult. At 16-17pcw in human, GluN2A-containing NMDA Rs are present in over 70% of cortical neurons, mainly within the cortical plate and subventricular zone [678]. It is not known how deficiency in the function of GluN2A-containing NMDA Rs might affect the ongoing formation of thalamocortical circuitry in the subplate, but overstimulation of glutamate Rs disturbs subplate structure and cortical plate lamination at E16.5 [683]. NMDAR activity in general is required for the correct formation of corticothalamic (CT)-thalamocortical (TC) circuitry. NMDA R antagonists compromise hippocampal neuronal migration and leading process extension in vitro [684], while germline deletion of the *Grin2a *gene in mice leads to miswiring of corticocortical fibres by P6 [587, 588]. Also, altered cortical structure in people with schizophrenia is associated with common *GRIN2A *variants [685]. Importantly, 7 T imaging of homozygous *Grin2a *deletion mice shows diffusion tensor imaging abnormalities in cerebral cortex and thalamus, tentatively interpreted as reduced axon density [686].

In addition, the neurite-outgrowth-promoting cytokines MDK and PTN, mentioned earlier as modulators of homeostatic plasticity that show strong genetic association with schizophrenia risk, appear to have an important function in the development of corticothalamic circuitry. Embryonically, *PTN *is expressed solely in cortical ventricular zone and prethalamus in mouse [687], suggesting a particular influence on thalamocortical circuit development. Indeed, PTN modulates TC tract guidance in the developing CNS, and promotes neurite outgrowth from E16 thalamic neurons in culture [589, 687, 688]. Although its actions are less well-characterised, MDK is extremely highly-expressed early in cortical development, with levels decreasing markedly in 3rd trimester [589, 687, 688]. This could explain the diminishing risk of offspring schizophrenia from prenatal maternal infection in 3rd trimester. Thus the influence of compromised synaptic plasticity to trigger the cascade of events leading to schizophrenia symptoms potentially acts early in brain development, around the time window associated with prenatal environmental risk. The risk variants may even exert a double action, introducing a functional frailty into thalamocortical circuits during development, and then impairing plasticity-mediated compensatory mechanisms during adulthood [9].

Considering that genetic data strongly implicate *ARC *in schizophrenia risk, it is important that there is direct evidence that mutations in Arc interactome genes interact with environmental factors to cause schizophrenia [689]. There is also suggestive evidence that environmental schizophrenia risk factors are linked to BDNF (and hence Arc and Narp). In pregnant mice and rats, maternal viral infection suppresses BDNF expression in embryonic brain, an effect maintained into offspring adulthood [690–694]. JNK is involved in the regulation of *Pvalb *expression by BDNF [320]. Indeed, we recently found that JNK is a key mediator of MIA effects on foetal brain [695]. Glucocorticoids (likely mediators of stress effects on foetus) can cross the placenta into the foetal compartment [696], and also suppress BDNF synthesis [621, 622, 697] and modulate cortical GABAergic interneuron gene expression, for example decreasing *Pvalb* and *Gad1 *expression [698]. Some complementary evidence suggests that prenatal maternal stress or glucocorticoid administration (in rats) can lead to lower BDNF levels in adult offspring brain [699, 700]. Hence one mechanism linking early developmental environmental influences to lasting plasticity deficits may be through impairment of BDNF function [9].

## Conclusions

It is not unequivocal that schizophrenia is associated with deficits in neuroplasticity. Direct assessment of the efficacy of plasticity processes in regions of the brain affected in the disease is not yet possible. However, while there is a lack of direct evidence for compromised synaptic plasticity in neural circuitry relevant to schizophrenia, the biochemical, pathological and genetic evidence is highly suggestive, as examined above. Schizophrenia may therefore usefully be viewed as a “neuroplastopathy”.

The following aspects merit emphasis:

1. All the neurobiological features that would be predicted to be present if plasticity was dysfunctional (as in impaired potentiation or inappropriate down-scaling) can be detected in schizophrenia. These include reduced spine density on glutamatergic neurones in PFC, reduced expression of *EGR1*, *NPTX2 *and* ARC* mRNAs and MAP2 protein in PFC, and metabolic hypofrontality. Further, the robustly observed reductions in PFC/HF *PVALB* and *GAD1* expression are entirely explicable in terms of chronically suppressed glutamatergic activity.
2. All forms and phases of post-synaptic plasticity are implicated by the genetic evidence. Genetic variants corresponding to initiating- (NMDA Rs, VDCCs), early- (ERK, PKA,PAK1 signalling pathways) and late- (*ARC*, *EGR1*) phase mediators of synapse-specific and homeostatic plasticity are all contributing to genetic risk.
3. Where the functional consequences of genetic risk variants are understood, the majority seem to be loss-of function mutations (e.g. rare mutations in NMDA R subunit genes, VDCC subunit genes and mutations that compromise the function of the αPIX/GIT2 complex [480], or TRIO [470], predicted to decrease ability to express LTP.
4. Mutations that facilitate plasticity have also been identified. For example, 16p11.2 (TAOK2/ERK1) duplications should enhance LTP. However, gain-of-function mutations in the Ras-Raf-Mek-Erk signalling cascade impair LTP as effectively as loss-of-function mutations [375], so potentially all genetic variants should be perceived as having an overall negative influence and disrupting the harmony of plasticity processes.
5. Chronic rather than acute NMDA R hypofunction appears to be crucial for modelling aspects of the disease in rodents. The evidence spans dendritic spine density in cortical neurons, γ oscillations, PFC metabolic activity, late-phase plasticity gene expression and *Pvalb *expression. This consistent pattern in the rodent studies is important because there is some evidence to suggest that the same is true in humans. Chronic ketamine abusers appear to show a range of symptoms that are even closer to those of schizophrenia per se than even the effects of acute ketamine [701]. This is most markedly the case for the negative symptom domains. Hence chronic NMDA R antagonist models should be preferred to acute models preclinically.
6. The rare, loss-of-function mutations in the *GRIN2A*gene conveying substantial schizophrenia risk seem to be recessive rather than dominant, with regard to their functional impact on the complete NMDAR R tetramer [399]. Since the rare mutation is hemizygous rather than homozygous, in most CNS NMDA Rs, the effect of the mutation will probably be overcome by the other fully functional GluN2 subunit present [702]. The mutation effect is probably only observed in diheteromeric NMDA Rs where the mutation is present in both GluN2A subunits (possible, in terms of cellular control of gene expression), or in triheteromeric NMDA Rs where the effect is not occluded by the other GluN2 subunit. This may provide some explanation for the relatively discrete neural circuitry affected in schizophrenia, despite the ubiquity of NMDA Rs, at least in such rare genetic cases.
7. Simply reducing network activity in mixed neuronal/glial cultures reproduces many of the neurobiological aspects of schizophrenia (reduced dendritic spine density, reduced *Bdnf*, *Arc*, *Nptx2* and *Egr1* expression, reduced *Pvalb*, *Gad1* and *Sst* expression, elevated TNFα expression) (Fig. 6). It is possible that an ability to normalise these changes in vitro might have value for initial screening of potential novel therapeutic approaches.

**Fig. 6:**
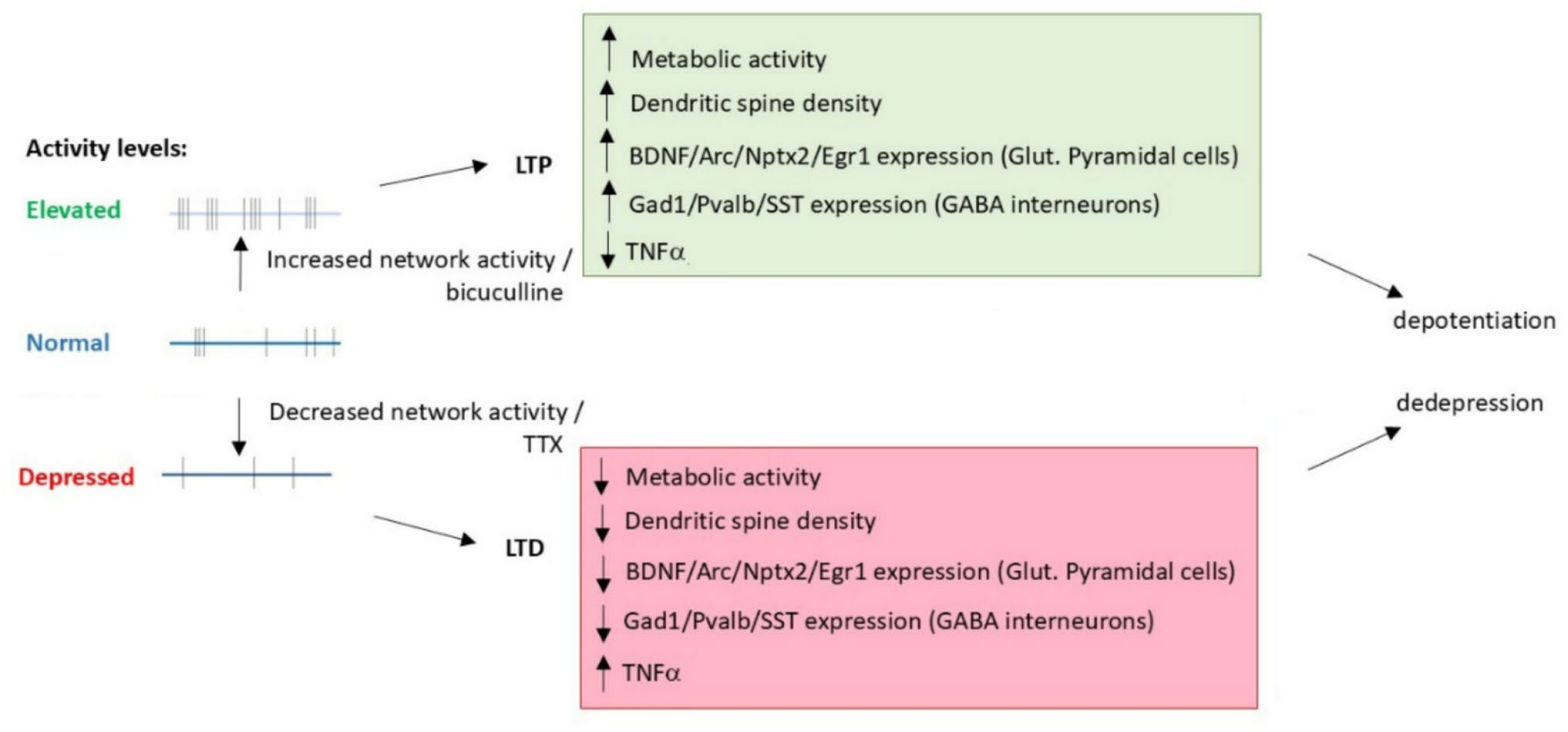
Neurobiological expression of synaptic plasticity in neuronal/glial mixed cultures, and similarities to the neurobiology of schizophrenia.
8. How environmental risk factors might contribute to a frailty in plasticity processes in a way that is selective for the neural circuitry affected is not known. Many of the genes/proteins described above are involved in neurodevelopment—neuronal migration, axon and dendrite growth, synapse formation—as well as plasticity in the mature brain. The cluster of environmental risk factors for schizophrenia early in development might suggest that this aspect of their function might be more relevant to understanding their influence in disease aetiology. Equally, aspects of the neurobiology of schizophrenia widely viewed as indicative of an early developmental disruption (loss of cortical dendritic spines, reduced *Pvalb *expression) are similarly explicable as products of impaired plasticity processes at a later stage. Moreover, it is hard to explain the regional selectivity of the loss of spines in PFC/HF through a generalised exposure of the foetal brain to the consequences of maternal infection, such as excessive microglial pruning. Perhaps the simplest explanation for the interaction of genetic and environmental risk factors might involve a fragility of corticothalamic circuitry present from its earliest formation, caused by adverse prenatal/perinatal environmental influences, that only becomes evident at the onset of overt symptoms when the circuitry matures and is put under stress [9]. The genetic factors could be seen as acting early in development, when the circuitry is forming, or in early adulthood when the frail circuitry is stressed.

Overall, it is clear that many of the neurobiological changes which are characteristic of schizophrenia (e.g. reduced PFC/HF metabolic activity, dendritic spine density and GABAergic or glutamatergic neuronal gene expression), can be interpreted as consequences of compromised plasticity processes. Furthermore, more than 60 of the genes potentially contributing to genetic risk are directly implicated in plasticity processes (including genes encoding receptors, voltage-sensitive Ca2^+^ channels, scaffold proteins, GTPases and kinases in signalling cascades). The hypothesis that a number of parallel mechanisms of plasticity are all likely to be implicated in the cascade of neurobiological events leading to schizophrenia symptoms is strongly supported by the available evidence. [10, 22, 28, 132, 158, 231, 703]

A key question that follows is how that hypothesis might inform the development of improved therapeutic strategies. Certainly small molecule agonists at the main BDNF receptor TrkB can be developed [704, 705], and these would be predicted to enhance PFC/HF *GAD1 *expression [320, 321] and facilitate increases in functional dendritic spines [706]. AMPA R facilitators might indirectly enhance BDNF expression [707]. Equally, effects of selective inhibitors of some of the kinases mediating LTD or depotentiation (Figs.3 and 4) remain relatively unexplored. Selective inhibitors of TNIK [708] and MAP4K4/HGK [709] remain little investigated in relation to plasticity processes. Mouse models of aspects of schizophrenia neurobiology with much strengthened translational validity are now available, and the phenotypes associated with disease-relevant plasticity impairment have been covered above and elsewhere [9]. The future prospects for identifying and testing (in vitro and in vivo) novel therapeutic strategies seem sufficiently strong to provide cause for optimism.

## Data Availability

No datasets were generated or analysed during the current study.
